# Protective Effects of the Ethanolic Leaf Extract of *Ocimum sanctum* L. on Collagen-Induced Human Platelet Aggregation and CCl_4_-Induced Hepatotoxicity in Rats: Molecular Docking Analysis of Platelet Activation Signaling Targets

**DOI:** 10.3390/molecules31162749

**Published:** 2026-08-07

**Authors:** Mohammad Maaz, Agilan Balupillai, Saravanabhavan Periyakali, Jasmin Padhan, Kumar Kalavathy Murugan, Joen-Rong Sheu, Jayakumar Thanasekaran

**Affiliations:** 1 Environmental Toxicology and Molecular Phytotherapy Lab, Department of Ecology and Environmental Sciences, School of Life Sciences, Pondicherry University, Puducherry 605014, India; mohammad.maaz2507@gmail.com; 2 Department of Biotechnology, School of Life Sciences, Pondicherry University, Puducherry 605014, India; agilanphd@gmail.com; 3 Department of Zoology, Bharathiar University, Coimbatore 641046, India; bhavan@buc.edu.in; 4 Department of Bioinformatics, School of Life Sciences, Pondicherry University, Puducherry 605014, India; jasminmgl@bicpu.edu.in (J.P.); kumarkm@bicpu.edu.in (K.K.M.); 5 Graduate Institute of Medical Sciences, College of Medicine, Taipei Medical University, Taipei 110, Taiwan

**Keywords:** *Ocimum sanctum* L., antiplatelet activity, CCl_4_-induced hepatotoxicity, oxidative stress, molecular docking, histopathology, GC/LC-MS

## Abstract

Natural products represent an important source of bioactive compounds with therapeutic potential in the fields of thrombotic and oxidative stress-related disorders. This study focuses on identifying active compounds from the ethanolic extract of *Ocimum sanctum* L. *leaves* (EEOSL) and validating its antiplatelet and hepatoprotective activities. GC-MS and LC-MS (both positive and negative ionization modes) analyses identified 155 and 113 compounds, respectively, constituting several bioactive compounds. Collagen-induced human platelet activation (CIHPA) and carbon tetrachloride (CCl_4_)-induced hepatotoxicity were used as rat models to assess the activity of EEOSL. EEOSL (1–10 mg/mL) was shown to significantly and dose-dependently inhibit collagen-induced platelet aggregation. The extract also inhibited P-selectin expression, ATP release and intracellular Ca^2+^ mobilization, suggesting inhibition of major pathways involved in platelet activation. To understand the mechanisms, molecular docking was carried out with key platelet signaling molecules such as GPVI, SYK, PLCγ2, P2RY12, and PI3Kβ. Some phytoconstituents showed good binding affinities, with apigenin having the highest binding affinity for PI3Kβ (−8.01 kcal/mol). This binding was further validated by molecular dynamics simulations showing the formation of stable complexes. Intraperitoneal injection of CCl_4_ significantly increased the serum hepatic markers (SGOT, SGPT, LDH and SALP); EEOSL treatment significantly reduced these increases in vivo. The extract normalized antioxidant enzyme activities (catalase, SOD and glutathione peroxidase) and antioxidant isozyme patterns. Histopathological results revealed a significant level of protection against liver damage. Overall, the results showed that EEOSL has phytoconstituents that could provide strong antiplatelet and hepatoprotective properties, likely due to their ability to modulate platelet signaling pathways and enhance antioxidant defense mechanisms.

## 1. Introduction

Platelets are small anucleated fragments of megakaryocytes that normally circulate in a dormant form. Upon the exposure of subendothelial collagen by vascular injury, platelet glycoprotein VI (GPVI) and integrin α2β1 receptors are activated, triggering intracellular signaling pathways that include the activation of phospholipase Cγ2 and the production of inositol 1,4,5 trisphosphate (IP_3_) [1]. Signaling through IP_3_ quickly releases intracellular Ca^2+^ stores, which are replaced by prolonged intracellular Ca^2+^ influx, which is another important platelet activation step [2]. There is an increase in cytosolic Ca^2+^, which leads to conformational activation of integrin α2β1, cytoskeleton rearrangement, and dense granule exocytosis, intensifying platelet aggregation [3]. The content of dense granules is released, leading to the extracellular release of ATP and ADP, which stimulate platelet recruitment by purinergic (P2) receptor signaling pathways [4]. At the same time, the α-granule secretion causes the platelet activation marker of P-selectin (CD62P) surface expression, which promotes platelet-leukocyte adhesion and includes thrombo-inflammatory mechanisms [5].

Therefore, intracellular Ca^2+^ mobilization, ATP release, and P-selectin expression are reputable biochemical evidence of platelet activation. Uncontrolled amplification of these pathways is tightly connected with thrombotic diabolical cardiovascular diseases, such as coronary artery disease and ischemic stroke [6]. However, even though the use of antiplatelet drugs like aspirin and P2Y12 receptor antagonists has proved effective in preventing thrombotic events, their long-term use has been linked to bleeding issues and hypersensitivity in some patients [7]. These restrictions indicate that safer, multitarget agents that have the capacity to mediate platelet signaling pathways are necessary. There is mounting evidence that platelet hyperreactivity is an important effect of oxidative stress. Reactive oxygen species (ROS) enhance Ca^2+^ signaling, granulocyte secretion, and P-selectin expression by activating redox-sensitive kinases and transcription [8,9]. Thus, antioxidant agents could reduce platelet activation through intracellular calcium homeostasis stabilization and secretory amplification.

The liver is the key metabolite and detoxifier of xenobiotics, and it is very prone to oxidative stress. Carbon tetrachloride (CCl_4_) is a popular hepatotoxin used in experimental studies, as its metabolic activation by cytochrome P450 enzymes leads to the formation of reactive trichloromethyl radicals, which cause lipid peroxidation and membrane damage [10,11]. This oxidative damage interferes with cell integrity and reduces the activity of endogenous antioxidant defenses such as superoxide dismutase (SOD), catalase (CAT), and glutathione peroxidase (GPx), which promotes hepatocellular necrosis and inflammation [12]. Oxidative stress is a common mechanistically related process in platelet activation and hepatic injury, and thus, agents that can restore redox balance can exert protective effects in either of these systems.

*Ocimum sanctum* L. is a medicinal plant that contains a lot of phenolic compounds, flavonoids, and essential oil constituents like eugenol and rosmarinic acid that have antioxidant and anti-inflammatory properties [13,14]. Such bioactive components have been said to counteract free radicals, suppress lipid peroxidation, and control redox-sensitive signaling pathways such as NF-κB and MAPK cascades [15,16]. Though the hepatoprotective effect of *Ocimum sanctum* on chemically induced liver injury has been previously reported [17], its direct effect on specified platelet activation parameters of intracellular Ca^2+^ mobilization, ATP release by dense granules, and P-selectin surface expression has not been thoroughly examined. Moreover, no study has so far given a simultaneous test on its impact on platelet activation by collagen and on oxidative hepatotoxicity by CCl_4_ under one mechanistic framework. Taking into consideration that oxidative stress is one of the most frequent pathological denominations between thrombotic diseases and liver injury, the measurement of agents that influence redox-dependent pathways in both settings is of great clinical interest. Thus, the current paper has explored the influence of the ethanolic extract of *Ocimum sanctum* leaves (EEOSL) on human platelet stimulation on collagen-stimulated platelets through the measurement of intracellular Ca^2+^ mobilization, ATP release, and the expression of P-selectin. To further validate the experimental data, molecular docking was performed to evaluate the binding affinity of the EEOSL major phytoconstituents against proteins involved in platelet activation signaling. Following this, molecular dynamics (MD) simulation was conducted for the top-ranked docked complex to evaluate its stability during the interaction. Moreover, the extracts’ hepatoprotective effects against CCl_4_-induced liver damage in rats were studied with a focus on antioxidant enzyme status and the histopathological changes involved.

## 2. Results

### 2.1. Chromatographic Profiling of EEOSL

**Figure 1:**
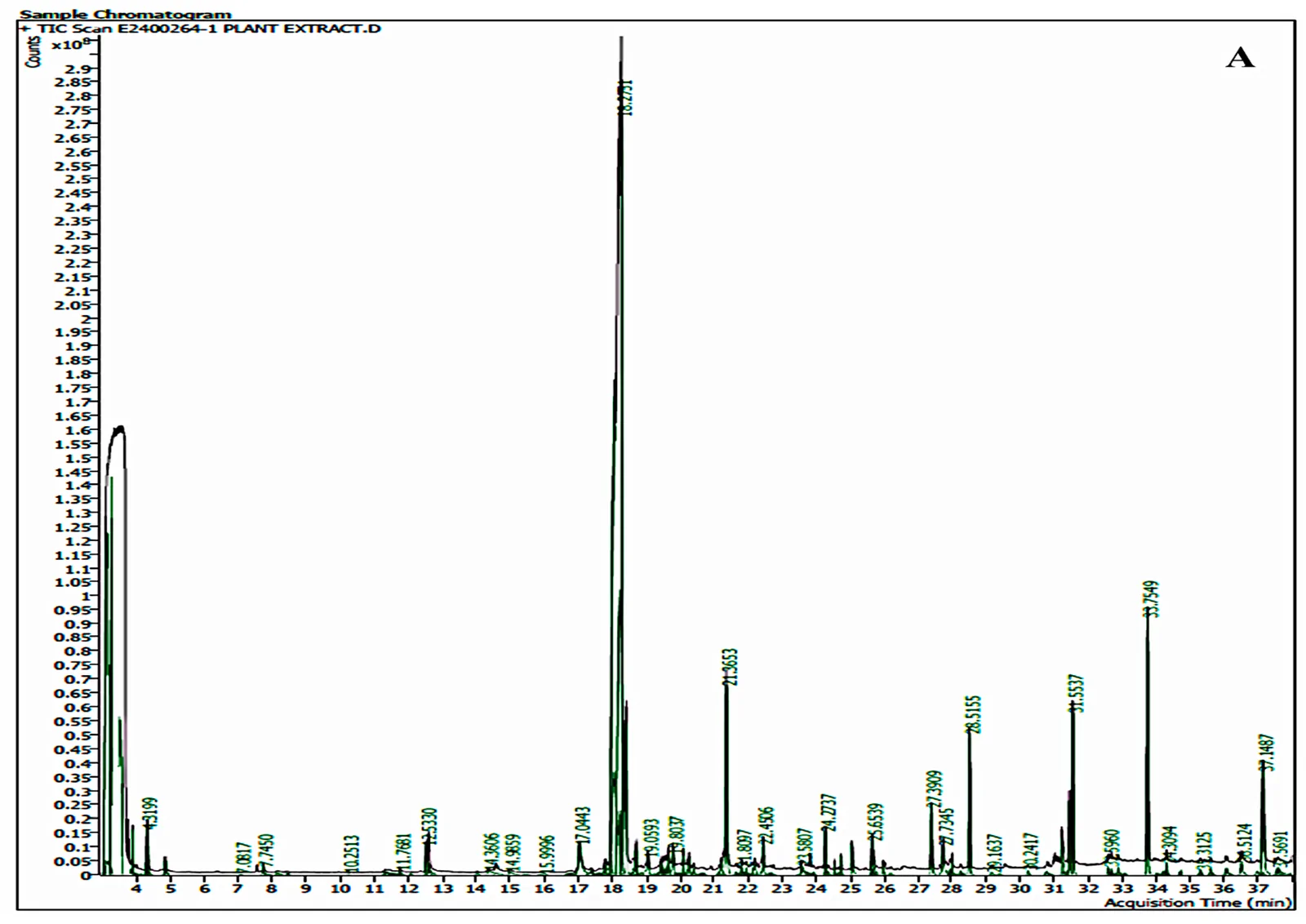

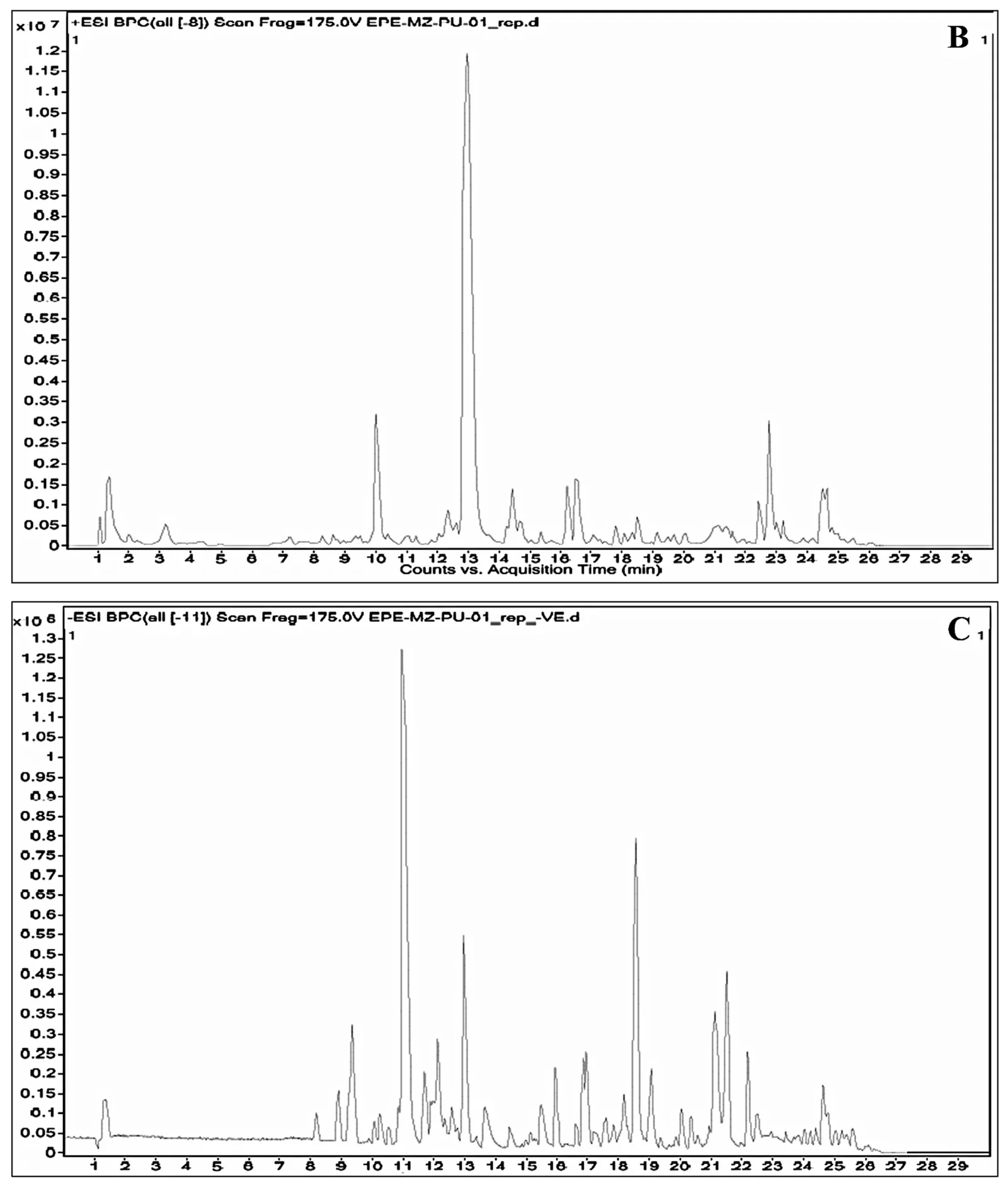
Chromatogram of EEOSL analyzed through (**A**) GC-MS, (**B**) LC-MS positive ESI, and (**C**) LC-MS negative ESI.

GC/MS screening revealed a total of 155 compounds. A detailed list of the identified phytoconstituents with their retention time and molecular formula is presented in the Supplementary Table S1. The chromatogram of the analysis is represented in Figure 1A. The phytoconstituents were matched with the NIST GC/MS library with the help of the total ion chromatogram (TIC) peaks. The need for a broader range of phytoconstituents present in the EEOSL led to the LC/MS screening. The analysis revealed a total of 113 compounds (including the compounds attained through both (+)ve and (−)ve ESI). The phytoconstituents identified with their retention time and molecular formula are represented in the Supplementary Table S2a,b and the chromatogram obtained can be seen in Figure 1B,C.

### 2.2. EEOSL Inhibits Collagen-Induced Human Platelet Aggregation

**Figure 2:**
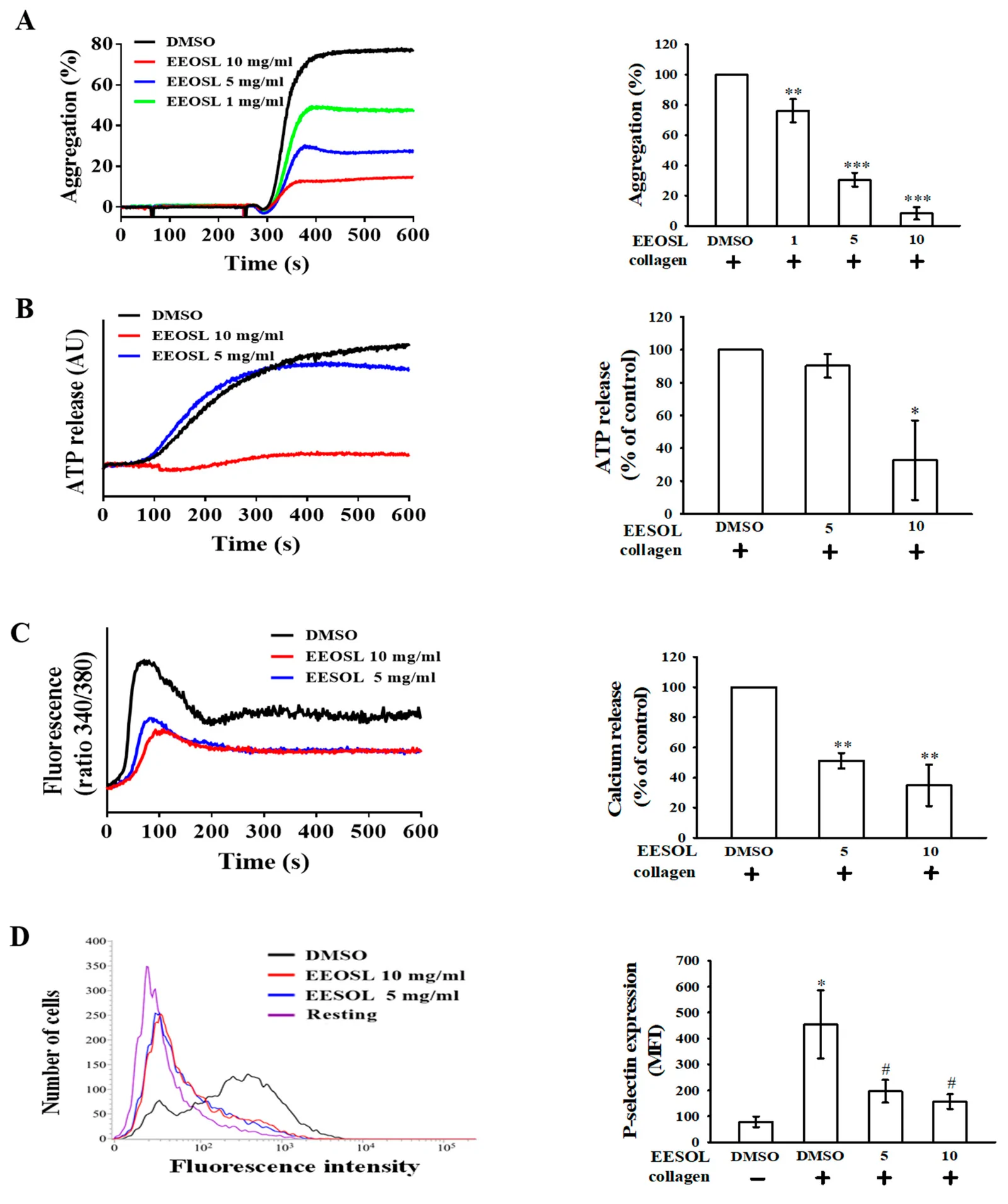
Effects of EEOSL on collagen-induced platelet activation. Washed human platelets (3.6 × 10^8^ cells/mL) were preincubated with a solvent control (0.1% dimethyl sulfoxide (DMSO) or EESOL (1–10 mg/mL) and subsequently treated with 1 μg/mL collagen. The effects that ensued were quantified in terms of (**A**) platelet aggregation, (**B**) ATP release, (**C**) relative [Ca^2+^]i mobilization, and (**D**) surface P-selectin expression. The data is presented in terms of the mean plus standard error of the mean (SEM) (*n* = 4). (**A**–**C**) * *p* < 0.05, ** *p* < 0.01, and *** *p* < 0.001 compared with the 0.1% DMSO + collagen group. (**D**) * *p* < 0.05 compared with the resting group; ^#^ *p* < 0.05 compared with the 0.1% DMSO + collagen group.

In the current study, EEOSL was found to greatly block platelet aggregation induced by collagen in human platelets. In the control group, a high response of platelets to stimulation was seen when collagen was used to stimulate the platelets. Nevertheless, pre-incubation of platelets in the presence of either 1 to 10 mg/mL EEOSL led to a significant and concentration-dependent decrease in aggregation (Figure 2A). EEOSL showed a fair inhibitory effect at lower concentrations (1 mg/mL), which reveals a partial block of collagen-mediated signaling pathways. The higher concentration of EEOSL (10 mg/mL) exhibited a significant inhibitory effect on platelet aggregation. This dose-dependent effect demonstrates that the antiplatelet action of EEOSL increases with the concentration.

### 2.3. EEOSL Inhibits ATP Release, Intracellular [Ca^2+^]i Levels and Surface P-Selectin Expression

To further clarify the antiplatelet effect of EEOSL, its influence on major indicators of platelet activation was also determined.

#### 2.3.1. Inhibition of ATP Release

An early step in platelet activation is dense granule secretion, which is an important step in platelet activation. Freed ATP and ADP serve as secondary mediators that enhance platelet activation through purinergic receptors (P2Y1 and P2Y12), which increase their aggregation. EEOSL dose-dependently inhibited the collagen-induced ATP release (Figure 2B). As the EEOSL concentration increased, the ATP secretion was inhibited, which revealed that the dense granule exocytosis was inhibited. This implies that EEOSL disrupts the amplification of pathways required for maintaining the platelet aggregation.

#### 2.3.2. Suppression of Intracellular [Ca^2+^] Mobilization

Calcium is an intracellular molecule that controls platelet activation through granule secretion, cytoskeleton restructuring, activation of phospholipase, and the activation of integrin αIIbβ3. Stimulation of collagen is usually followed by a rapid increase in the level of calcium in the cell cytoplasm, which occurs due to the release of the intracellular stores and the entry of calcium into the cells. EEOSL at both concentrations tested had a significant effect on the elevation of intracellular [Ca^2+^] caused by collagen (Figure 2C). This observation suggests that EEOSL can disrupt the upstream signaling events (including activation of phospholipase C, production of IP_3_, activation of calcium channels, etc.), thus impairing the calcium-sensitive platelets.

#### 2.3.3. Suppression of Surface P-Selectin Expression

P-selectin is held in the α-granules and, during activation, translocates to the platelet surface. Surface P-selectin expression is a well-known hallmark marker of platelet activation and has a significant contribution to platelet-leukocyte interaction and thrombus stabilization. The P-selectin surface expression was significantly enhanced by collagen stimulation. EEOSL, however, strongly inhibited this upregulation (Figure 2D), which implies that it inhibited α-granule secretion. This observation also supports the fact that EEOSL inhibits platelet activation at the secretory responses level. A standard antiplatelet medication (aspirin or clopidogrel) was not part of the present study; however, future studies will directly compare EEOSL to clinically proven antiplatelet medications to further delineate the relative efficacy.

### 2.4. Molecular Modeling Studies

Initial screening used molecular docking to estimate how well 15 phytochemicals from EEOSL might bind to enzymes involved in platelet activation. Each compound was docked against all five target proteins, and the resulting Glide SP scores for all the targets were compared to identify the highest predicted binding affinity. Lower (more negative) scores suggest a tighter fit (as summarized in Table 1). The docking scores of all the compounds against all the targets have been included in the Supplementary Table S3.

**Figure 3:**
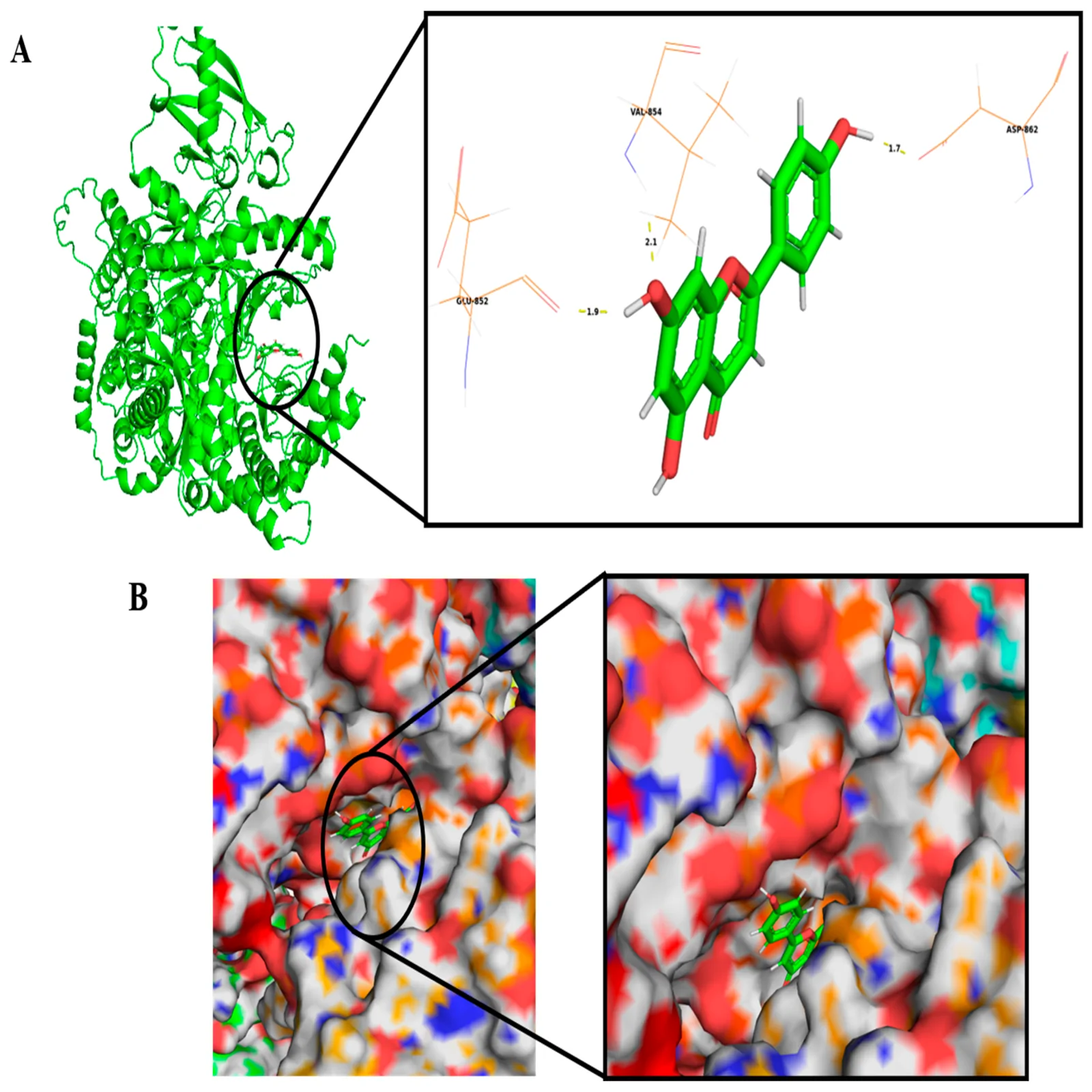
(**A**) The 3D map shows how apigenin interacts inside the catalytic pocket of PI3Kβ. The image highlights important non-covalent contacts and outlines both the hydrogen-bonding network and significant hydrophobic interactions with nearly all residues; (**B**) detailed 3D binding orientation of apigenin captured within the PI3Kβ receptor cavity. Apigenin is shown in a stick model, clearly highlighting the spatial arrangement of the nearby amino acids that helps with the binding event.

Among the ligands studied, apigenin stood out with the strongest binding affinity in the entire study, scoring −8.01 kcal/mol against PI3Kβ (UniProt: P42338). When we looked at the pose, apigenin fit snugly inside the active site by forming three distinct hydrogen bonds with Glu852, Val854, and Asp862. This shows stable accommodation of the ligand within the active site (Figure 3A,B). The other proteins also had their best matches but with weaker scores: 3-O-acetylpadmatin docked to GPVI (PDB: 2GI7) at −4.61 kcal/mol, methyl salicylate to SYK (PDB: 4FL2) at −3.53 kcal/mol, isoferulic acid to P2RY12 (PDB: 4NTJ) at −6.60 kcal/mol, and kaempferol-3-glucuronide bound to PLCγ2 (PDB: 8T7C) at −5.84 kcal/mol. The spectrum of the bioactive compounds having the best docking score and the two-dimensional (2D) protein–ligand interactions is presented in the Supplementary Figures S1–S6. These computational insights suggest that several compounds in the extract likely interfere with distinct points in the platelet signaling network. However, since the PI3Kβ-apigenin pairing produced such a notably strong score, we prioritized it as our main candidate for MD simulations.

### 2.5. Molecular Docking of Platelet Activation Signals

To ensure that the docking pose of the PI3Kβ-apigenin complex was not just a static artifact, we conducted a 100 ns molecular dynamics simulation. We measured RMSD, RMSF, H-bond, and Rg to assess the structural stability of the complex in a simulated aqueous environment.

**Figure 4:**
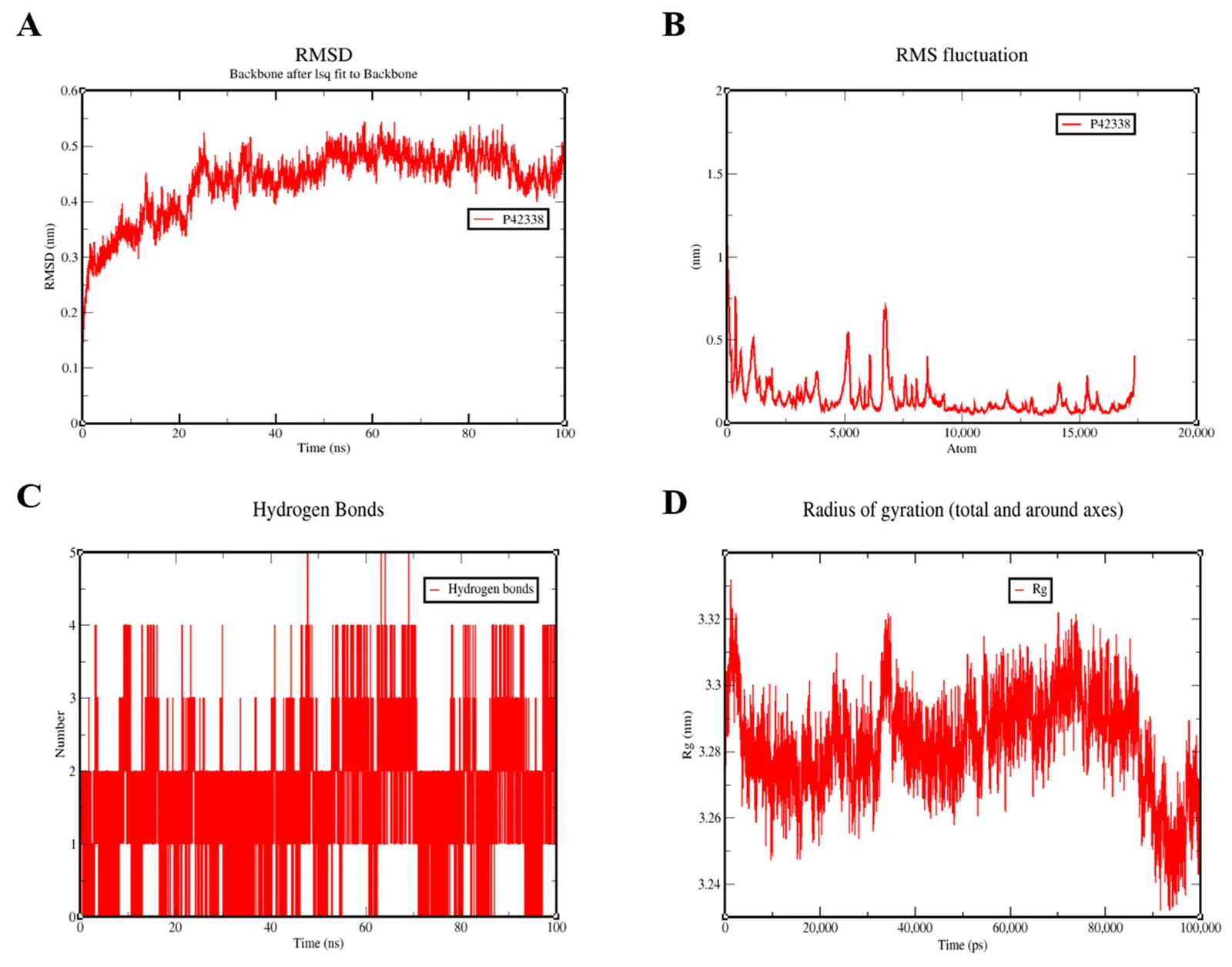
Molecular dynamics simulation analyses showing the physical behavior of the PI3Kβ-apigenin complex during a 100 ns simulation. (**A**) The backbone RMSD curve demonstrates the system’s structural equilibration and stabilization over time. (**B**) The RMSF scatter plot highlights the localized flexibility of individual protein residues. (**C**) The H-bond tracking indicates consistent, uninterrupted molecular interactions between the ligand and the active site. (**D**) The Rg plot measures the protein’s overall compactness, confirming that the complex kept its folded shape without expanding or collapsing during the simulation.

Tracking the backbone RMSD showed an initial rise from 0.20 nm to about 0.35 nm as the protein adjusted to the solvent, indicating early structural changes after solvation and equilibration. However, by the 25 ns mark, the curve flattened out between 0.45 and 0.50 nm. It remained stable in that range for the rest of the run, demonstrating that the overall structure of the PI3Kβ-apigenin complex reached a stable state (Figure 4A).

The local flexibility analysis through RMSF showed that most of the protein backbone barely moved, fluctuating by less than 0.2 nm. A few isolated peaks reached 0.5–0.7 nm, but mapping these fluctuations onto the 3D structure confirmed that they were just loose terminal ends and surface loops, leaving the core binding pocket intact (Figure 4B).

The H-bond analysis revealed that apigenin maintained between 1 and 4 hydrogen bonds with PI3Kβ during the simulation, briefly spiking to 5 hydrogen bonds at times. This consistent bonding pattern demonstrates the stability of the complex, as the ligand did not drift out of the pocket; it remained firmly docked throughout the entire 100 ns period (Figure 4C).

Finally, the Rg barely shifted, staying steady between 3.24 and 3.32 nm from start to finish. Since the Rg did not suddenly jump or drop, it indicates that the protein did not unfold or swell abnormally when apigenin bound to it (Figure 4D). Overall, these metrics strongly support the docking results, confirming that apigenin forms a dynamically stable complex with PI3Kβ.

### 2.6. Effect of EEOSL on Serum Enzyme Parameters

In this experiment, rats of Group II treated with carbon tetrachloride (CCl_4_) showed higher serum level of SGOT, SGPT, SALP, and LDH compared to the normal control group (Group I). CCl_4_ is a proven hepatotoxicant that causes hepatotoxicity by the formation of reactive metabolites like trichloromethyl (•CCl_3_) and trichloromethyl peroxy (•OOCCl_3_). These radical species trigger lipid peroxidation, membrane disruption of hepatocytes and eventually lead to the release of intracellular enzymes into the bloodstream.

**Figure 5:**
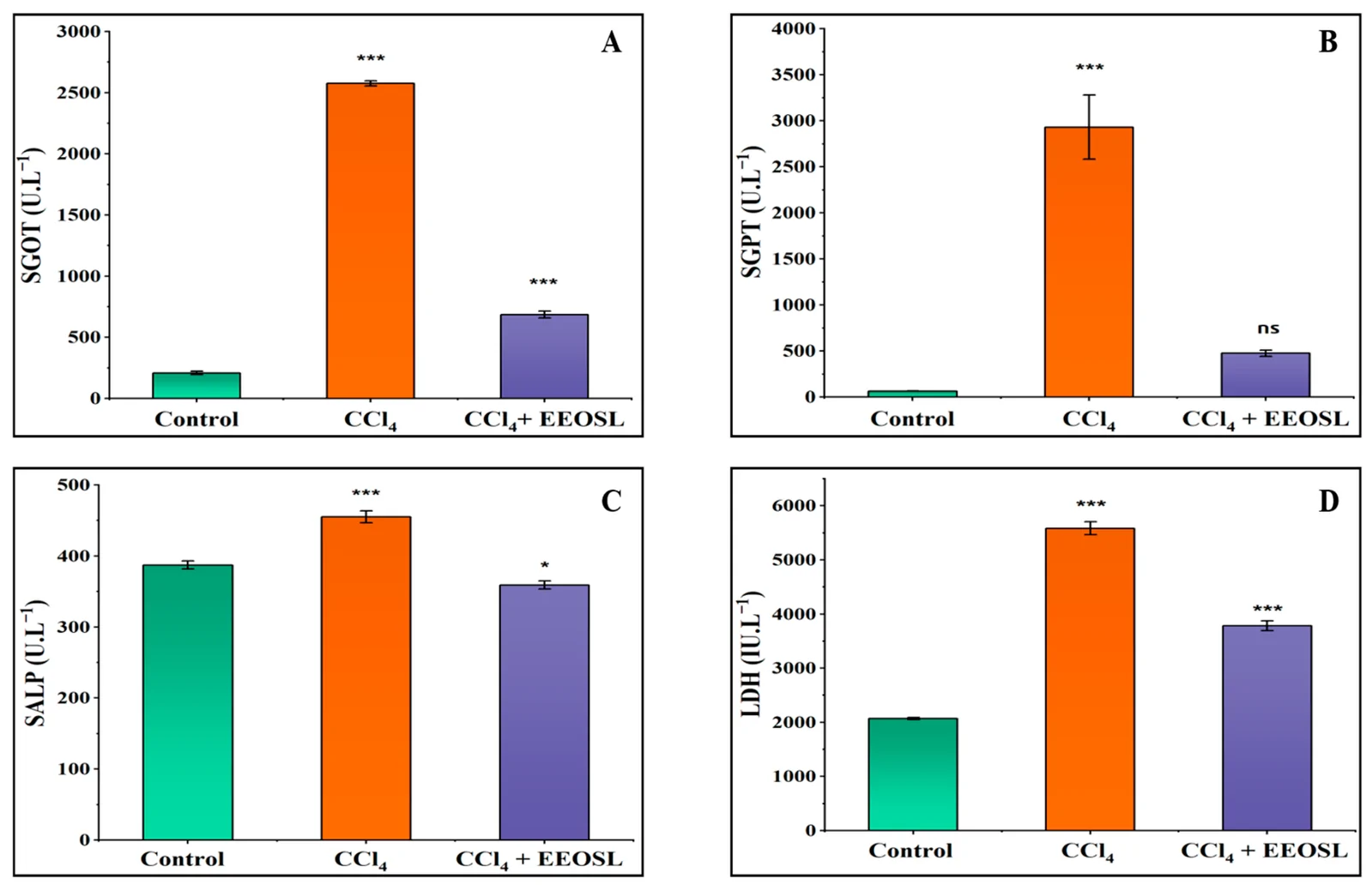
EEOSL effect on serum liver biomarker enzymes in CCl_4_-induced liver injury in Wistar rats. The levels of (**A**) serum glutamic oxaloacetic transaminase (SGOT), (**B**) serum glutamic pyruvate transaminase (SGPT), (**C**) serum alkaline phosphatase (SALP), and (**D**) lactate dehydrogenase (LDH) were determined in Wistar rats with CCl_4_-induced liver damage. The results are in the form of mean ± standard error of the mean (*n* = 5). * *p* < 0.05, *** *p* < 0.001, a non-significant difference compared to the control group is denoted by ns.

Treatment of Group III (CCl_4_ + EEOSL), however, significantly reduced the enzyme levels back to normal (Figure 5A–D). The high decrease in SGOT and SGPT levels indicates the reduction in hepatocellular injury, and the restoration of normal SALP levels is an indicator of better biliary function and membrane stabilization. Similarly, a drop in LDH activity indicates a drop in cellular necrosis and an increase in tissue integrity. It has been shown that serum enzyme levels in EEOSL-treated rats were restored, indicating its hepatoprotective effect, which could be due to its antioxidant activity, free radical scavenging activity, and stabilization of hepatocyte membranes. The results indicate that EEOSL can be used to prevent the hepatotoxic effects of CCl_4_ and preserve normal liver function.

### 2.7. EEOSL Enhances CCl_4−_ Mediated Reduction in Antioxidant Enzymes

In the current experiment, the hepatic SOD, CAT, and GPx activities were significantly lowered in rats of Group II (CCl_4_-treated) relative to the normal control group (Group I) (Table 2). The fact that these antioxidant enzyme activities were reduced shows that the oxidative stress was acute, caused by the administration of CCl_4_. Nonetheless, EEOSL treatment in Group III (CCl_4_ + EEOSL) raised the SOD, CAT, and GPx activities to near-normal levels. The increase in the activity of SOD indicates better degradation of superoxide radicals into hydrogen peroxide. The increase in CAT and GPx activity demonstrates that there are better processes of hydrogen peroxide and lipid peroxide detoxification, and that oxidative harm to the hepatic tissue is decreased.

### 2.8. EEOSL Regulates the Isoenzyme Profile of Antioxidant Enzyme in CCl_4_-Induced Liver in Rats

#### 2.8.1. Effects on CAT Isozyme

**Figure 6:**
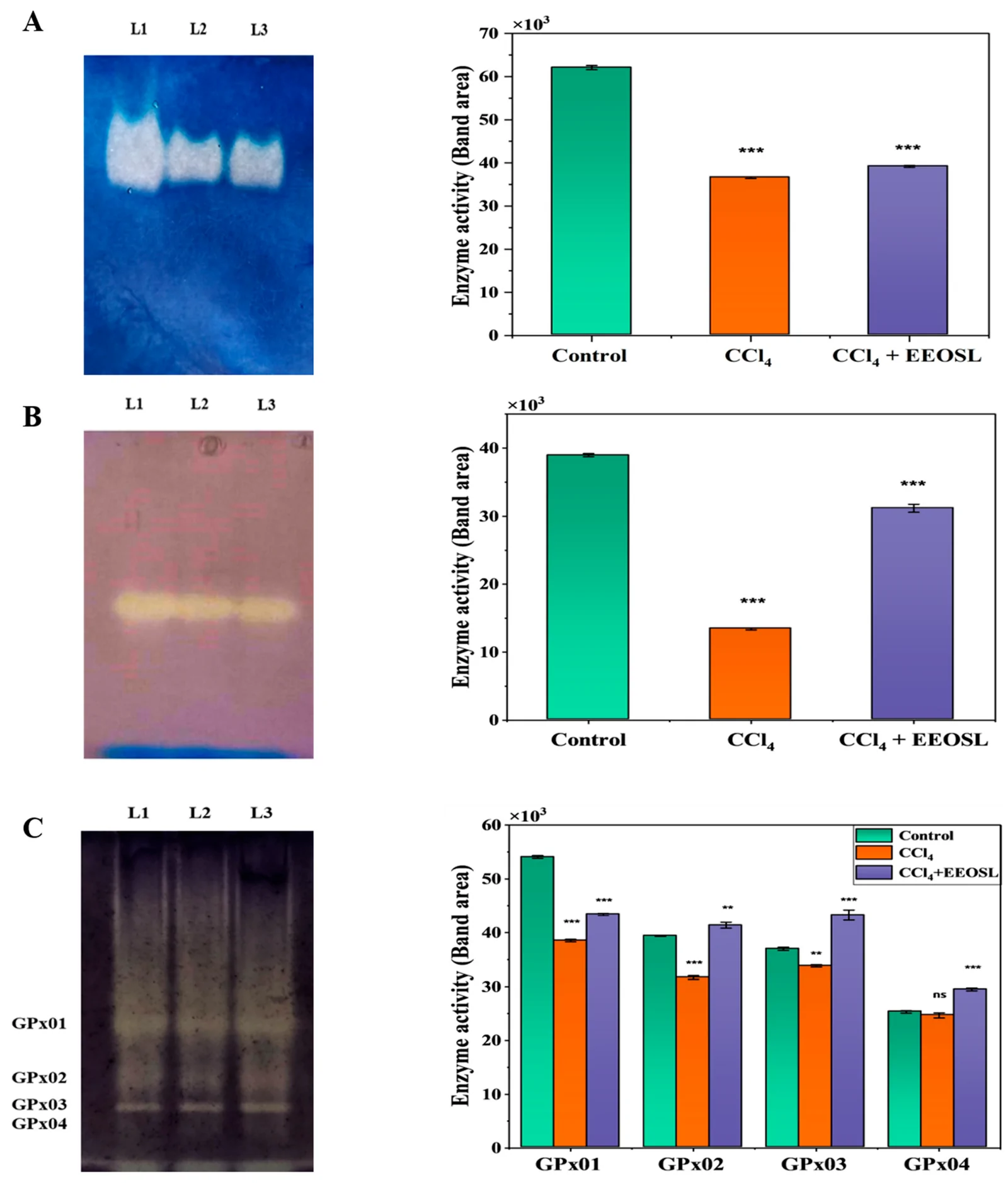
EEOSL influences the antioxidant enzymes catalase (**A**), superoxide dismutase (**B**) and glutathione peroxidase (**C**) isozyme in liver tissue of rats induced with CCl_4_. Native-PAGE was used to measure catalase (CAT), superoxide dismutase (SOD) and glutathione peroxidase (GPx) isozyme in liver tissue. (**top panel**) Representation gel picture of L1 (normal), L2 (treated with CCl_4_) and L3 (treated with CCl_4_ + EEOSL). (**bottom panel**) Densitometric pattern of isozyme activity in liver tissue. The results are in the form of mean ± standard error of the mean (*n* = 5). ** *p* < 0.01, *** *p* < 0.001, a non-significant difference compared to the control group is denoted by ns.

Considering the liver homogenates of all three experimental groups, the results of non-denaturing electrophoretic analysis showed one CAT isozyme band that was detectable (Figure 6A), which implies that no other isozyme variants were activated under the experimental conditions. Nevertheless, the intensity of the bands differed significantly among the groups, revealing differences in the activity and expression level of the CAT enzyme. Group I, the normal control, had a high-intensity CAT band with a band area of 62,107.2, which indicates a good defense against physiological stress conditions. In contrast, Group II rats that received only CCl_4_ had a significantly smaller band intensity (band area: 36,597.48), indicating a marked depletion or inactivation of CAT due to CCl_4_-induced oxidative stress. The decreased staining intensity indicates a reduced ability to deactivate hydrogen peroxide, thereby increasing oxidative injury in hepatic tissue. Interestingly, Group III rats (CCl_4_ + EEOSL treatment) showed a significant recovery of CAT band intensity (band area: 39,198.91) compared with that of the CCl_4_ group (Figure 6A). Although only partial recovery was observed and the intensity did not reach that of the normal control group, the results indicate that EEOSL is effective in reducing oxidative stress and improving CAT enzyme stability or expression.

#### 2.8.2. Effects on SOD Isozyme

In this study, all the experimental groups showed only one SOD isozyme protein band (Figure 6B), indicating that no other isoforms were induced by the administration of CCl_4_ and EEOSL treatment. It was, however, found that there were significant differences in band intensity among the groups, resulting in differences in enzyme activity and expression. Group I had a strongly stained SOD band with a band area of 38,968.13, which indicated normal antioxidant capacity. On the same note, Group III rats (CCl_4_ + EEOSL) had one of the highest band intensities, with a band area of 31,177.95. Though slightly lower than the control group, the intensity was still significantly higher than that of the CCl_4−_ only group.

Conversely, the band intensity in Group II rats that were exposed to CCl_4_ alone was significantly lower, and the band area was 13,431.11 (Figure 6B). Such a tremendous decrease indicates severe depletion or inactivation of SOD by overwhelming oxidative stress caused by the CCl_4_ metabolites. Reduced SOD levels would increase superoxide radical generation, which would further promote lipid peroxidation and hepatocellular injury.

#### 2.8.3. Effects on GPx Isozyme

All three experimental groups showed four different GPx isozymes (Figure 6C), which indicated that there were a variety of GPx forms expressed in the hepatic tissue. A major difference in the intensity of the band staining was, however, found between the groups. The four GPx isozymes were easily observed in the normal control group (Group I) and their staining was moderate, indicating normal antioxidant capacity. Conversely, the staining intensity of the four GPx isozymes in Group II rats treated with CCl_4_ alone showed a significant reduction (Figure 6C). This reduction implies that there was oxidative inactivation or down-regulation of the GPx enzymes caused by the overproduction of free radicals and lipid peroxidation induced by CCl_4_. This reduced GPx activity would hinder the process of peroxide detoxification and thus increase the level of hepatic oxidative injury.

Interestingly, compared with Group I rats (control group), Group III rats (CCl_4_ + EEOSL treatment) had an increase in the intensity of all GPx isozymes except GPx01. The higher activity of the GPx isozymes in EEOSL-treated rats indicates the stimulatory or protective action on the expression and stability of antioxidant enzymes. This increase could indicate the mobilization of endogenous antioxidant defense mechanisms and improved redox homeostasis in the hepatic tissue.

### 2.9. Effects of EEOSL on CCl_4_-Induced Histological Alterations

To determine the structural changes in the liver tissues of the experimental groups, histopathological examination of liver tissues was conducted using hematoxylin and eosin (H&E) staining according to the experimental groups.

**Figure 7:**
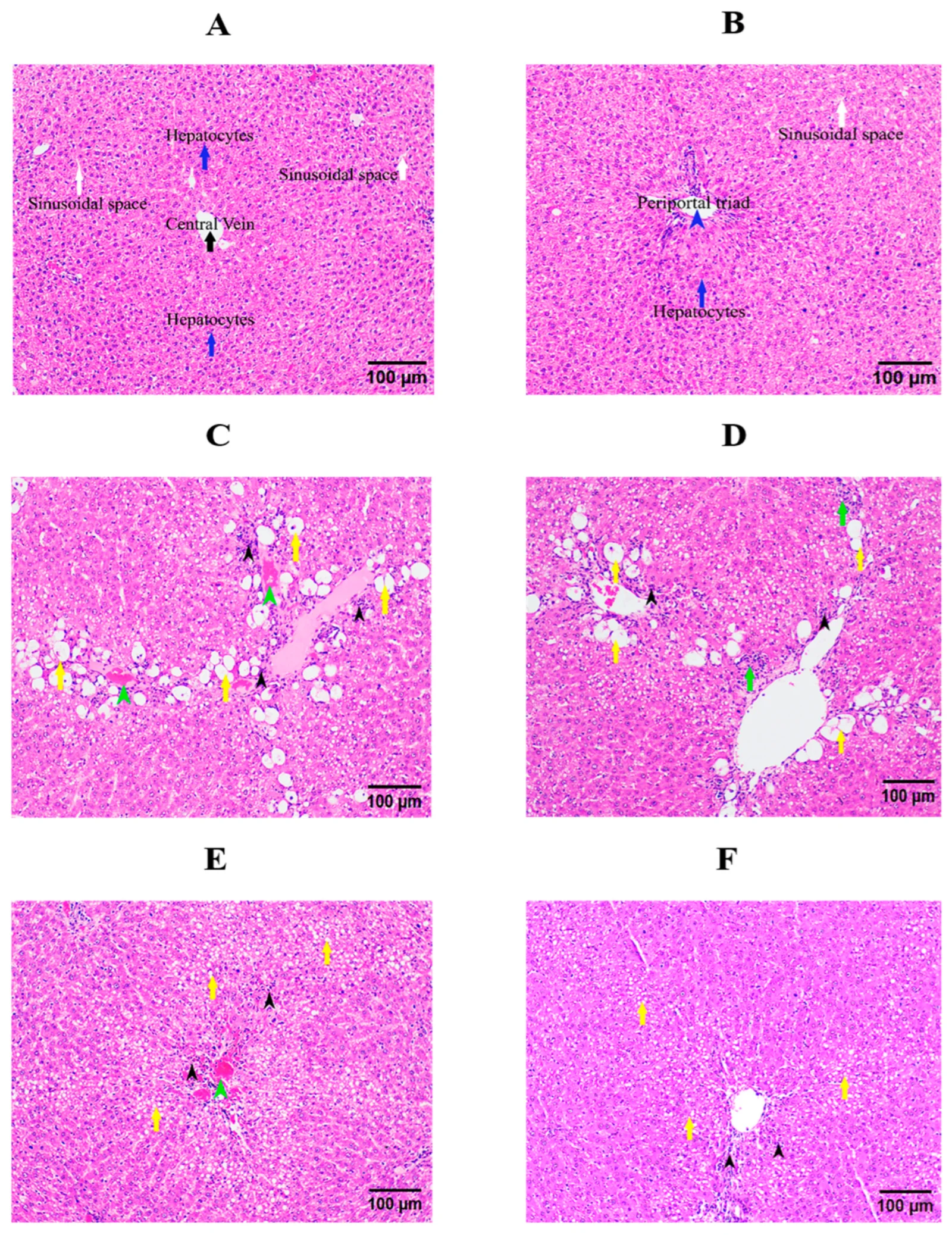
Photomicrographs of liver tissue sections of the control, CCl_4_-treated and EEOSL-treated rats. Photomicrographs of H & E-stained liver tissue sections depicting (**A**,**B**) the control, (**C**,**D**) the carbon tetrachloride (CCl_4_)-treated, and (**E**,**F**) the CCl_4_ + EEOSL (500 mg/kg b.w.)-treated are given at a magnification of 100× (scale bar = 100 μm). In panel (**A**,**B**), blue arrows mark normal hepatocytes, white arrows mark sinusoids, black arrows mark the central vein, and blue pointed arrows mark the portal triad. The presence of severe diffuse vacuolation in the hepatic cytoplasm is indicated by yellow arrows in panel (**C**,**D**), perivascular inflammation by black pointed arrows, congestion by green pointed arrows, and multifocal necrosis by green arrows. In panels (**E**,**F**) the yellow arrows represent moderate diffuse cytoplasmic vacuolation of the hepatic cytoplasm, the black pointed arrows represent perivascular inflammation, and the green pointed arrows represent congestion.

Sections of the liver from the normal control (Group I) displayed an intact hepatic architecture with properly organized hepatocytes having a typical polygonal form, centrally positioned prominent nuclei, and distinct plasma membranes. The hepatic lobular structure remained undisturbed with central veins appearing normal and sinusoidal spaces well defined. The portal vein, hepatic artery, and bile duct, which formed the periportal triad, were also found to be structurally normal, indicating the preservation of vascular and biliary integrity (Figure 7A,B). In comparison, liver sections of Group II rats that received CCl_4_ exhibited severe pathological changes. Diffuse cytoplasmic vacuolation, hepatocellular ballooning, inflammatory infiltration in the peripheral areas, vascular congestion and foci of necrosis were observed (Figure 7C,D). Such structural defects are typical of CCl_4_-induced hepatotoxicity and are indicative of extensive oxidative injury, membrane disruption, and inflammatory reactions in hepatic tissue.

Nonetheless, liver sections of Group III (CCl_4_ + EEOSL treatment) showed significant improvement in histological architecture when compared to the CCl_4_ alone group. Despite the fact that moderate cytoplasmic vacuolation, mild perivascular inflammation, and congestion were still evident, the hepatic architecture as a whole did not seem to be significantly affected. Hepatocytes maintained near- normal morphology, and the structural composition of hepatic cords and sinusoids was significantly restored (Figure 7E,F). These histological results support the biochemical and enzymatic results that EEOSL has a protective effect on CCl_4_-induced hepatic injury.

## 3. Discussion

In recent years, plants have been extensively explored for their therapeutic and pharmacological activities. Owing to the presence of diverse bioactive constituents, medicinal plants serve as valuable sources for commercial drug development [18]. The current study aimed to screen the phytoconstituents of the EEOSL using chromatographic techniques and to investigate the combined effect of EEOSL as both an antiplatelet and hepatoprotective agent. Overall, the findings indicate the presence of a wide array of phytoconstituents in the extract and a significant modulatory role of the extract in thrombosis and hepatic injury.

The chromatographic analyses of the extract revealed an array of bioactive constituents, contributing to its therapeutic efficacy. Some major ingredients identified included eugenol, methyleugenol, 2,4- di-tert-butylphenol, β-caryophyllene, phytol, tocopherols, caffeic acid, ascorbic acid, apigenin, methyl salicylate, kaempferol-3-glucuronide, 3-O-acetylpadmatin, isoferulic acid, quercitrin, and azelaic acid. All these compounds have been known to impart wide-pharmacological properties, like antioxidant, hepatoprotective, anti-inflammatory, anticancer, antimicrobial, and neuroprotective activities [19,20]. The antiplatelet study demonstrated that treatment with EEOSL reduced collagen-induced platelet aggregation. Platelet activation can be triggered by various agonists [21]. Collagen, the agonist used in this study, plays a pivotal role in platelet activation by binding to the glycoprotein VI (GPVI) receptor on platelets. During vascular injury, platelets adhere to the exposed subendothelial matrix component, collagen, forming a complex that acts both as an adhesive surface and a signaling stimulus, thereby initiating platelet activation. Treatment with EEOSL showed significant, dose-dependent inhibition of collagen-induced platelet aggregation, suggesting its effect on platelet–platelet interaction and the phospholipase C-mediated signaling pathway [22].

Platelet activation initiates a cascade involving tyrosine kinase signaling, leading to increased intracellular calcium concentration and exocytosis of granules such as P-selectin and ADP/ATP. ATP functions as a secondary agonist, recruiting additional platelets, while elevated intracellular calcium levels trigger morphological changes, granule secretion, and aggregation, thereby promoting thrombus growth. P-selectin, stored in α-granules, is translocated to the platelet surface upon activation. The release of α-granules during exocytosis is a hallmark of platelet activation and is commonly assessed by measuring P-selectin expression [23]. In the present study, EEOSL dose-dependently inhibited ATP release and impaired calcium mobilization. Furthermore, a reduction in P-selectin expression was observed. Additionally, molecular docking was performed to validate the role of EEOSL phytoconstituents in the platelet activation process. The bioactive phytocompounds that were docked showed promising activity against the collagen-induced GPVI/SYK/PLCγ2 and ADP signaling proteins P2RY12/PI3Kβ with the PI3Kβ-apigenin complex showing the most favorable binding, with a binding score of −8.01 kcal/mol. Further, evaluation of the PI3Kβ-apigenin complex through MD simulations confirmed its stability. Collectively, these findings indicate that EEOSL modulates both the early and late phases of platelet activation. These observations are consistent with previous reports [22,23] demonstrating that eugenol and methyl eugenol, two important constituents of *Ocimum* species, suppress collagen-induced platelet activation.

The liver is a vital organ responsible for numerous biological functions, including metabolism, detoxification, and digestion [24]. Liver diseases account for approximately 4% of global deaths annually (1 in 25 deaths worldwide) [25]. Hepatic disorders can arise through multiple mechanisms, including exposure to environmental pollutants. Acute liver injury is closely associated with oxidative stress, which leads to the generation of reactive oxygen species (ROS). These free radicals impair liver function by creating an imbalance between oxidants and the antioxidant defense system. Consequently, oxidative stress contributes to hepatic damage characterized by inflammation, vasodilation, cellular hypertrophy, bile duct proliferation, fibrosis, and necrosis [26]. Various experimental models have been employed to study drug-induced liver injury [27]. In the present study, CCl_4_-induced hepatotoxicity was used as the experimental model. CCl_4_ is a well-known hepatotoxin that induces liver damage primarily through oxidative stress. The resulting free radical formation leads to leukocyte infiltration, vascular occlusion, and excessive collagen deposition, ultimately causing severe hepatic injury [26]. Modulation of oxidative stress is thought to be a convergence point for the dual biological activities of EEOSL. ROS enhance platelet activation by increasing intracellular calcium signaling and also increase lipid peroxidation and hepatocellular injury induced by CCl_4_, which further amplifies platelet activation. Thus, the antiplatelet and hepatoprotective properties observed might share a common mechanism, which is redox homeostasis.

The natural products have been one of the most reliable sources of bioactive compounds with varied pharmacological effects and, in particular, antioxidant potential. Many clinically useful medications such as quinine, morphine, and paclitaxel have their source in plants [28]. The Lamiaceae family is a cosmopolitan family that contains a large variety of medicinally valuable plants. *Ocimum sanctum* (also referred to as Holy Basil or Tulsi) is one of them and has been used conventionally in a number of therapeutic systems due to its antioxidant, anti-inflammatory, anticancer, anti-ulcer, and antimicrobial properties [14]. The therapeutic effect of *Ocimum sanctum* is believed not only to be due to individual phytochemicals but also to the interaction between these phytochemicals. Moreover, the phytochemical profile of *O. sanctum* varies as a function of cultivar, geographic origin, environmental conditions, and extraction processes, so a detailed phytochemical characterization is necessary. Therefore, the elucidation of the chemical properties and biological activities of *O. sanctum* has been an attractive point of interest and can be used in its future development and as a source of drugs, even if there are similar compounds in other medicinal plants.

Biochemical indicators expressed in serum are sensitive indicators of liver dysfunction. Liver damage causes hyperpermeability of the cell membrane and hepatocellular injury, causing the leakage of intracellular enzymes, including SGPT (ALT), SGOT (AST), SALP (ALP), and LDH into the bloodstream [29]. These serum enzymes increased significantly in rats treated with CCl_4_ in the current study, proving hepatocellular damage. Nevertheless, EEOSL treatment was also associated with the significant decrease in the serum levels of these enzymes, which demonstrated the stabilization of hepatocyte membranes and recovery of liver function. The results are in line with the previous literature that demonstrated the hepatoprotective property of plant-based antioxidants [30,31]. CCl_4−_ induced hepatotoxicity is mainly mediated via oxidative stress. When activated metabolically in the liver, CCl_4_ produces extremely reactive free radicals, which cause lipid peroxidation, protein oxidation, and cell membrane damage. Thus, interventions rich in antioxidants are a potential method to reverse oxidative stress- induced liver damage. The liver has a strong antioxidant enzyme defense system, such as catalase (CAT), superoxide dismutase (SOD), and glutathione peroxidase (GPx). These enzymes act in synergy to counter the ROS. SOD catalyzes the breakdown of superoxide radicals into hydrogen peroxide, which is later neutralized into water and oxygen by CAT and GPx. Disruption of this antioxidant defense mechanism causes excess accumulation of ROS, which eventually causes hepatic dysfunction [24].

In the present study, CCl_4_ treatment had a great impact on reducing the activity of CAT, SOD, and GPx, which supports the findings from earlier studies [24,30,32]. The reduction in antioxidant enzyme activity is a sign of excessive oxidative stress and the weakening of the cellular defenses. It is worth noting that EEOSL treatment restored the activities of these enzymes to normal levels, indicating that the extract has a strong antioxidant effect. This restoration suggests that EEOSL can directly scavenge free radicals or increase the expression of endogenous antioxidant enzymes, which in turn protects hepatocytes against oxidative damage caused by CCl_4_. Biochemical results were further supported by histopathological analysis. In Group II (CCl_4_ treated) rats there was severe disruption of the architecture of the liver tissues with cell degeneration and structural disorganization. Group III (CCl_4_-exposed rats treated with EEOSL) on the other hand showed significantly better hepatic architecture with little structural change and very close to the control group (Group I). These results confirm the protective effect of EEOSL at the tissue level. Similar hepatoprotective effects of natural products in a CCl_4_ model have been shown by previous studies. Normal hepatocyte architecture was restored by the methanolic extract of *Pleurospermum candollei* with clear nuclei [24]. There was a dose-dependent reduction in inflammatory cell infiltration and hepatocyte destruction in mice treated with CCl_4_ by Shibi tea extract [30]. The aqueous extract of *Carica papaya* seeds enhanced hepatocellular regeneration and reduced fatty acid-related changes [33]. Similarly, *Pleurotus ostreatus* (oyster mushroom) with antioxidant properties resorted the hepatocyte architecture and sinusoidal structure of following liver damage due to CCl_4_ in Wistar albino rats [32]. This pattern of protection, as detected in the current study, is in line with these results, which serve to support the therapeutic potential of antioxidant-rich natural products. Thus, the biochemical and histopathological changes in CCl_4_-treated rats were markedly attenuated by the use of EEOSL, which indicates a high degree of hepatoprotective activity. Its antioxidant properties may explain the protective effect, counteracting oxidative stress, preserving cellular integrity, and restoring normal hepatic architecture. All these results underscore the therapeutic potential of EEOSL as a natural antioxidant agent in the treatment of oxidative stress-induced liver diseases.

In the light of the present findings, we suggest a unified model in which polyphenols and flavonoids obtained from EEOSL act by reducing oxidative stress, and thereby inhibit platelet activation and hepatic damage. The decrease in oxidative stress reduces calcium mobilization, ATP secretion, and P-selectin expression in platelets, which results in decreased platelet aggregation. In the liver, increased activity of antioxidant enzymes helps to prevent lipid peroxidation and maintain the integrity of hepatocytes. Oxidative stress modulation is, therefore, the common mechanism underlying both pharmacological activities.

## 4. Materials and Methods

### 4.1. General Experimental Procedure

All the reagents and chemicals used in the experiment were of analytical grade. Fresh leaves of *Ocimum sanctum* Linn. were gathered from the area of Pondicherry University, India, and were verified by Dr. N. Parthasarathy, Professor, Pondicherry University. The leaves collected were dried under shade for one week and roughly powdered. The powdered material was extracted using a Soxhlet apparatus with ethanol as the solvent. A rotary evaporator was used to concentrate the resulting crude extract under reduced pressure and it was then stored at 4 °C until further use.

### 4.2. GC-MS Analysis of EEOSL

The methodology utilized by Srivastava et al. [34] was followed for the GC-MS analysis of EEOSL, with slight modifications. The Agilent CH-GCMSMS02, 8890 GC, 7000 GC/TQ (Agilent Technologies, Santa Clara, CA, USA) system equipped with an HP5MS column of dimensions 30 m × 250 µm × 0.25 µm was used for the analysis. The carrier gas used was helium with a constant flow rate of 1.0 mL/min. The column temperature was initially set at 50 °C and held for 2 min, followed by an increase to 120 °C for 16 min. Again, the temperature was increased to 210 °C for 10 min and finally to 280 °C for 12 min, resulting in a total run time of 38 min. The scan range was 30–900 *m*/*z*, and the solvent delay was 3 min. A sample volume of 2 μL was injected into the system.

### 4.3. LC-MS Analysis of EEOSL

The study followed the methodology described by Venuprasad et al. [35], with some modifications. The analysis was performed on the HRLCMS-QTOF system (Agilent Technologies, Santa Clara, CA, USA) using the data acquisition software Agilent Mass Hunter, Version Q-TOF B.05.01 (B5125.3). The column used was a Hypersil GOLD C18 (100 mm × 2.1 mm, particle size 3 μm). The solvent conditions were (A) 0.1% formic acid in Miili-Q water and (B) acetonitrile operated in gradient mode: (i) 95% A/5% B from 0 to 1 min (starting condition), (ii) 100% B from 1 to 25 min (maximum organic phase), (iii) 100% B from 26 to 30 min (holding at maximum organic phase), (iv) 95% A/5% B from 30 to 31 min (re-equilibration started) and (v) 95% A/5% B from 31 to 35 min (back to the initial condition). The flow rate was 0.3 mL/min, the injection volume was 5 μL, and the ESI parameters were as follows: both negative and positive ionization mode; mass range: 120–1200 m/z; spray voltage: 3.5 kV; gas temperature: 250 °C; gas flow rate: 13 L/min; and nebulizer pressure: 35 psi.

### 4.4. Platelet Preparation

Blood samples were obtained from f healthy volunteers who had not taken any non-steroidal anti-inflammatory drugs (NSAIDs) within two weeks before the study. As an anticoagulant, whole blood was collected in polypropylene tubes with acid citrate/dextrose solution (ACD; 9:1, *v*/*v*). The collected blood was centrifuged at 120× *g*, and platelet-rich plasma (PRP) was obtained after centrifugation for 10 min. The PRP layer was thoroughly aspirated and incubated with heparin and prostaglandin E1 to avoid platelet activation. Then, PRP was centrifuged at 500× *g*, and the platelet pellet was collected after 10 min. The pellets were washed and resuspended twice in Tyrode’s solution containing bovine serum albumin (BSA, 3.5 mg/mL). Calcium chloride was added to obtain a final Ca^2+^ concentration of 1 mM. This was brought up to a final platelet concentration of 3.6 × 10^8^ cells/mL. The study protocol for human platelet experiments was approved by the Institutional Review Board of Taipei Medical University (TMU-JIRB-N202112047).

### 4.5. Platelet Aggregation Assay

The turbidimetric technique using a luminometric aggregometer (Payton, Scarborough, ON, Canada) was used to measure platelet aggregation. EEOSL (1–10 mg/mL) and the vehicle (0.1% DMSO) were pre-incubated with washed platelet suspensions (3.6 × 10^8^ cells/mL) for 3 min. Platelet aggregation was triggered by the addition of collagen (1 μg/mL). The aggregation response was recorded for an additional 6 min and expressed as the percentage aggregation compared with the control.

### 4.6. ATP Release and Calcium Mobilization Assay

A luciferase/luciferin bioluminescence assay was used to determine the ATP release. Intracellular calcium mobilization was measured using the fluorescent calcium indicator Fura-2 AM. Fluorescence signals (Ca^2+^ mobilization), luminescence (ATP release) were measured on a Hitachi F-7000 Fluorescence Spectrophotometer (Tokyo, Japan), as per the manufacturer’s instructions. The intracellular calcium concentration was determined by the fluorescence intensity ratio at an excitation wavelength of 340/380 nm.

### 4.7. P-Selectin Expression

Flow cytometry was used to analyze the surface expression of P-selectin. Washed platelets were incubated with an FITC-conjugated anti-P-selectin monoclonal antibody (2 μg/mL) and EEOSL (5 and 10 mg/mL) for 3 min. After preincubation, collagen (1 μg/mL) was used to induce P-selectin expression. Platelets labeled with FAM were identified by a BD FAC Scan (Becton Dickson, San Jose, CA, USA). Each sample was analyzed using 10,000 platelets. Gating and identification of platelets were done according to the characteristic light profiles of forward scatter (FSC) and side scatter (SSC). The findings were expressed as surface P-selectin expression measured as mean fluorescence intensity (MFI).

### 4.8. Molecular Docking

The target proteins selected for molecular docking were glycoprotein VI (GPVI; PDB ID: 2GI7), spleen tyrosine kinase (SYK; PDB ID: 4FL2), phospholipase Cγ2 (PLCγ2; PDB ID: 8T7C), and the purinergic receptor (P2RY12; PDB ID: 4NTJ). The respective three-dimensional (3D) structures were retrieved from the RCSB Protein Data Bank (RCSB PDB: https://www.rcsb.org/) on 1 April 2026, along with phosphoinositide 3-kinase β (PI3Kβ; UniProt ID: P42338), obtained from the UniProt database (https://www.uniprot.org/). These proteins were selected because they represent key components of platelet activation pathways involved in collagen and ADP-mediated signaling.

Molecular docking was performed using the Schrödinger suite (Schrödinger Release 2025-1: Glide, Schrödinger, LLC, NY, USA, 2025) [36,37,38]. Protein structures were prepared using the Protein Preparation Wizard by removing non-essential water molecules, adding missing side-chain atoms, assigning appropriate bond orders and protonation states, and minimizing the structures to relieve steric clashes [39]. Ligands were prepared using the LigPrep module of Maestro (Schrödinger Release 2025-1: LigPrep, Schrödinger, LLC, NY, USA, 2025), which generated low-energy three-dimensional conformations with appropriate stereochemical, protonation, and ionization states.

**Figure 8:**
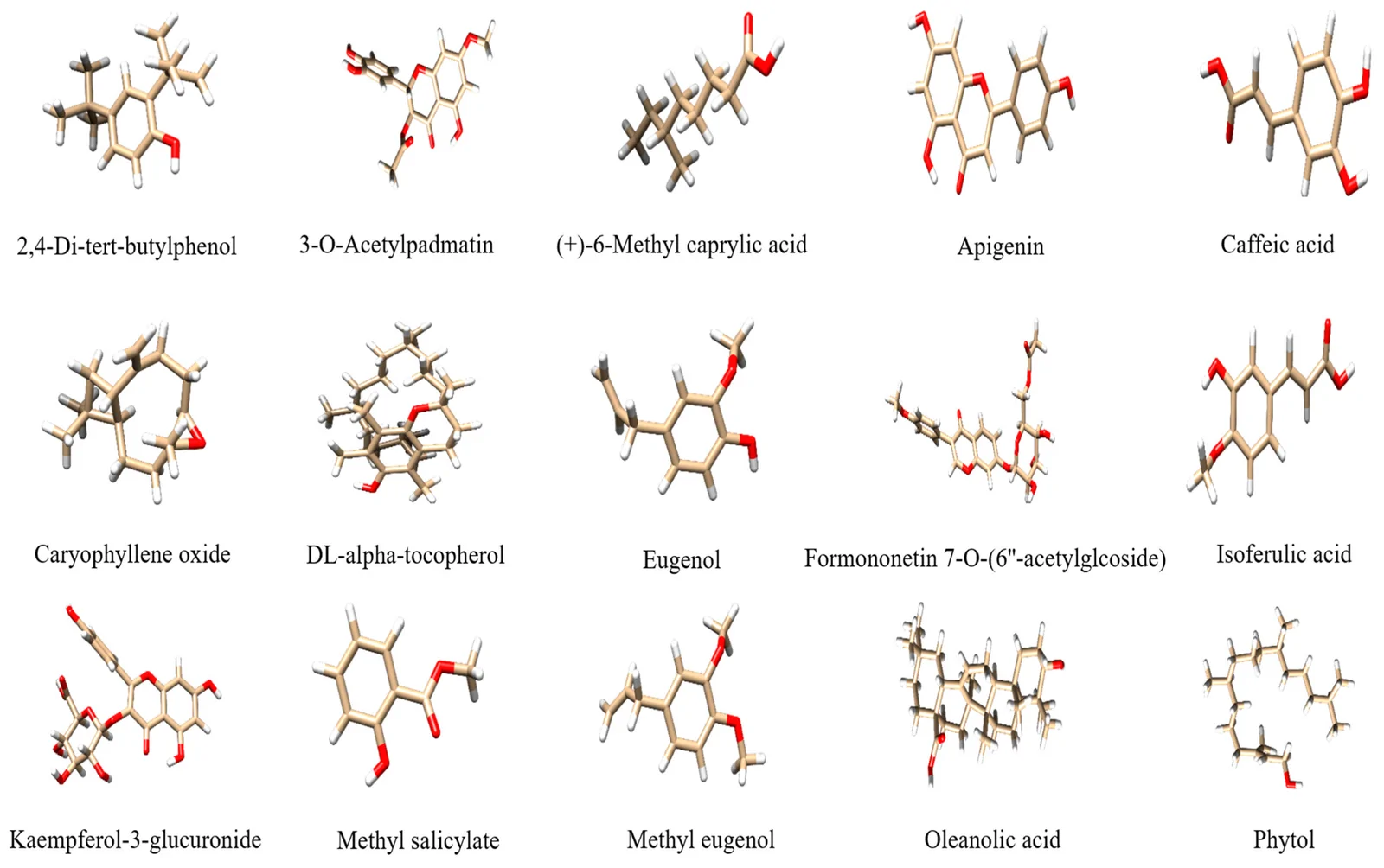
Spatial 3D chemical structures of the fifteen unique phytoconstituents evaluated in the molecular docking study.

Receptor grids were generated based on the known ligand-binding region of each target protein, and binding-site prediction was further supported using the COACH-D 2.0 server [40]. The active-site residues used for docking are listed in Table 3, and the structures of the selected ligands are shown in Figure 8. Flexible ligand docking was then carried out using the standard-precision (SP) Glide algorithm [41] and docking scores together with protein–ligand interaction patterns were used to identify the most promising complexes for further molecular dynamics analysis.

### 4.9. Molecular Dynamics Simulation

Molecular dynamics (MD) simulation of the selected protein–ligand complex was performed using GROMACS version 2023.4 [42,43] with the CHARMM27 (Version: c32b1) force field (CHARMM22 plus CMAP correction for proteins) [44]. The topology and parameters for the apigenin ligand, compatible with the CHARMM force field, were generated using the SwissParam web server [45]. The complex was placed in a triclinic simulation box using the TIP3P water model. A minimum distance of 1.0 nm was maintained between the solute and the box boundaries using the GROMACS editconf module. The solvated system was neutralized by replacing solvent molecules with Na^+^ and Cl^−^ ions using the gmx genion module.

Energy minimization was carried out using the steepest descent algorithm to remove steric clashes and unfavorable contacts. The system was then equilibrated under the NVT and NPT ensembles with positional restraints of 1000 kJ mol^−1^ nm^−2^ applied to the complex. Therefore, the restraints were removed and let the production simulation was run for 100 nanoseconds (ns). The structural stability and dynamic behavior of the complex were examined by analyzing the backbone root mean square deviation (RMSD), root mean square fluctuation (RMSF), radius of gyration (Rg), and intermolecular hydrogen bonds (H-bonds) using standard GROMACS analysis tools.

### 4.10. Animal Experiments

Male healthy Wistar rats (180–250 g) were purchased from the Biogen Laboratory Animal Facility, Bangalore, India. The animals were housed at the Central Animal House Facility, Pondicherry University, in a controlled environment (25 ± 2 °C; 12 h light/dark cycle). Animals were permitted to acclimatize for 14 days, after which the experiments were conducted with normal pellet food and water ad libitum. Three groups of (*n* = 5) rats were randomly selected: Group I (normal control) received the vehicle (olive oil, 1 mL/kg body weight) for four days. Group II (toxin control) received the vehicle on day one and day four; on day two and day three, CCl_4_ (50 % *v*/*v* in olive oil, 2 mL/kg b.w.) was administered to the rats. Group III (test group) received EEOSL (500 mg/kg b.w.) on days 1 and 4; on days 2 and 3, the rats received both EEOSL and CCl_4_. All the treatments were delivered through the intraperitoneal route. Animals were sacrificed on day 5. Blood samples were obtained through the retro-orbital plexus, and liver tissues were collected immediately. Serum and liver samples were frozen at −80 °C for subsequent biochemical analyses. The experimental procedures involving rats were approved by the Institutional Animal Ethics Committee (IAEC), Pondicherry University (Approval No. PU/CAHF/30th IAEC/2025/04), and all experiments were carried out strictly in accordance with the CPCSEA (Committee for the Purpose of Control and Supervision of Experiments on Animals) guidelines.

### 4.11. Determination of Serum Liver Marker Enzymes

Serum liver enzymes were measured as biochemical markers of liver function. Serum biochemical indicators (SGPT (ALT), SGOT (AST), SALP (ALP), and LDH) were measured as per standard laboratory procedures. The enzyme activities were reported in U/L (SGPT, SGOT, SALP) and IU/L (LDH).

### 4.12. Preparation of Rat Tissue Homogenate

Liver tissue (100 mg) was homogenized in 1 mL of 50 mM phosphate buffer (pH 7.0) under ice-cold conditions. The homogenate was centrifuged at 10,000 rpm for 15 min at 4 °C. The supernatant was collected for biochemical analyses. The amount of total proteins was determined according to the standard procedure [46].

### 4.13. Quantitative Analysis of Antioxidant Enzymes

The activities of hepatic antioxidant enzymes were determined spectrophotometrically using a double-beam UV–Visible spectrophotometer (Model: LMSPUV1900, LABMAN Scientific Instruments Pvt. Ltd., Chennai, Tamil Nadu, India). The activities of superoxide dismutase (SOD), glutathione peroxidase (GPx), and catalase (CAT) were determined in accordance with the guidelines proposed by Marklund and Marklund [47], Rotruck et al. [48] and Sinha [49], respectively.

### 4.14. Native-PAGE Isozyme Detection

Antioxidant isozymes were detected using Native-PAGE. Native-PAGE was performed according to the procedure described by Laemmli [50]. Liver homogenates were prepared without any denaturing agents, such as SDS and samples were not boiled before electrophoresis. Equal amounts of proteins (20 µg) were loaded onto the gels. The separation of SOD and GPx isozymes was performed on 10% polyacrylamide gels, and CAT isozyme separation was done on 8% gels. Electrophoresis was performed at 4 °C using a fixed voltage of 50 V (stacking gel) and 80 V (separating gel) using the Mighty Small II Mini Vertical Electrophoresis Unit (Model SE250-10A-.75, Hoefer Inc., San Francisco, CA, USA). Isozymes’ specific staining was performed according to the procedures followed by Beauchamp and Fridovich [51], Lin et al. [52] and Woodbury et al. [53] for SOD, GPx, and CAT, respectively.

### 4.15. Histopathological Examination

Histopathological examination of liver tissues was performed using conventional paraffin-embedding and hematoxylin and eosin (H & E) staining with slight modifications [54]. At the end of the experimental period, the rats were euthanized under CO_2_ euthanasia, and necropsy was performed. The livers were carefully excised and fixed in 10% neutral buffered formalin for tissue preservation. The fixed tissues were cut into small sections (4 mm) and kept overnight under running tap water or remove formalin. Following this process, tissue processing was performed using graded concentration of alcohol (50%, 70% and 90%; absolute alcohol 1 and 2) for dehydration and xylene for clearance, followed by embedding in60 °C molten paraffin. Tissue sections were cut using a rotary microtome at a thickness of 4 µm and the dried slides were stained with H & E, where hematoxylin, a basophilic stain, imparts a blue color to the nuclei, whereas eosin, an acidophilic stain, imparts a pink color to the cytoplasm. The stained slides were mounted with DPX mounting medium. Finally, the mounted slides were dried and examined under a light microscope (Lynx, Lawrence & Mayo India Pvt. Ltd., Kolkata, West Bengal, India).

### 4.16. Statistical Analysis

The experimental results are presented as the mean ± standard error of the mean (SEM). One-way analysis of variance (ANOVA) with Tukey’s honestly significant difference (HSD) post hoc test was conducted in OriginPro 2026 (Version 10.3.0.197, SR1; OriginLab Corporation, Northampton, MA, USA) to determine the statistical significance of the differences. A *p*-value t < 0.05 was regarded as statistically significant.

## 5. Conclusions

EEOSL demonstrated strong antiplatelet and hepatoprotective properties by reducing the platelet activation responses and enhancing the endogenous antioxidant defense system. The extract inhibited collagen-induced platelet aggregation, ATP release, and intracellular calcium mobilization, and also prevented CCl_4_-induced liver damage by restoring antioxidant enzyme activities and maintaining liver histoarchitecture. Through molecular docking, various phytoconstituents were found to show potential binding to important platelet signaling proteins, with apigenin having the greatest binding affinity towards the PI3Kβ protein. The stability of the PI3Kβ–apigenin complex was also confirmed by molecular dynamics simulation. The results indicate that the protective effects of EEOSL could be attributed to modulation of platelet signaling as well as oxidative stress pathways as the main mechanisms. However, additional target-based validation, pharmacokinetic investigations, and clinical trials will be needed to validate the therapeutic relevance of apigenin and other active constituents.

## Figures and Tables

**Table 1:** Molecular docking scores (kcal/mol) of the top-ranked phytoconstituents against key platelet activation proteins.

Sl. No.	Protein	Protein ID	Ligand	Compound ID (CID)	Docking Score (kcal/mol)
1	GPVI	PDB: 2GI7	3-O-Acetylpadmatin	10406203	−4.61
2	SYK	PDB: 4FL2	Methyl salicylate	4133	−3.53
3	PLCγ2	PDB: 8T7C	Kaempferol-3-glucuronide	22846027	−5.84
4	P2RY12	PDB: 4NTJ	Isoferulic acid	736186	−6.60
5	PI3Kβ	UniProt: P42338	Apigenin	5280443	−8.01

**Table 2:** Effects of EEOSL on the activities of catalase (CAT), superoxide dismutase (SOD) and glutathione peroxidase (GPx) in the liver tissue of rats with CCl_4_-induced injury.

Antioxidant Enzymes	Group of Animals		
	Group-I (Control)	Group-II (CCl_4_-Induced)	Group-III (CCl_4_ + EEOSL)
CAT	705.06 ± 9.722	224.12 ± 13.253 ***	615.02 ± 8.055 ***
SOD	1.46 ± 0.005	0.71 ± 0.011 ***	0.92 ± 0.007 ***
GPx	1.48 ± 0.005	0.69 ± 0.005 ***	1.03 ± 0.002 ***

Values are expressed as mean ± standard error of mean (*n* = 5); *** *p* < 0.001 compared with control CAT- µmoles of H_2_O_2_ utilized/min/mg protein SOD- unit/mg protein GPx- µmoles of GSH oxidized/min/mg protein.

**Table 3:** Target proteins and their predicted active-site residues used for molecular docking.

Protein	Active Site
GPVI (PDB ID: 2GI7)	L35, R37, E39, Y46, R66, Q70, W75
SYK (PDB ID:4FL2)	S299, G300, N301, F302, V305, A320, K322, V353, M368, E369, M370, A371, P375, R418, N419, L421, D432, Y445, K453
PLCγ2 (PDB ID: 8T7C)	N328, E357, D359, E406
P2RY12 (PDB ID: 4NTJ)	F85, Y86, M89, Y90, I155, F160, F206, F210, F213, H214, L245, S249
PI3Kβ (Uniprot ID: P42338)	M779, P785, W787, I803, K805, D813, Y839, I851, V853, V854, S857, D923, M926, I936, D937

## Data Availability

The original contributions presented in this study are included in the article/Supplementary Materials. Further inquiries can be directed to the corresponding authors.

## Supplementary Materials

The following supporting information can be downloaded at: https://www.mdpi.com/article/10.3390/molecules31162749/s1, Figure S1, Regular and zoomed spectrum of 3-O- Acetylpadmatin identified from HR LC-MS analysis of EEOSL; Figure S2, Regular spectrum of Methyl salicylate identified from GC- MS/MS analysis of EEOSL; Figure S3, Regular and zoomed spectrum of Kaempferol 3-glucuronide identified from HR LC-MS analysis of EEOSL; Figure S4, Regular and zoomed spectrum of Isoferulic acid identified from HR LC-MS analysis of EEOSL; Figure S5, Regular and zoomed spectrum of Apigenin identified from HR LC-MS analysis of EEOSL; Figure S6, Two-dimensional (2D) protein–ligand interaction diagrams generated using “Ligand Preparation” option of the Maestro, illustrating the binding interactions of (A) apigenin with PI3Kβ (UniProt: P42338), (B) 3-O-acetylpadmatin with GPVI (PDB ID: 2GI7), (C) methyl salicylate with SYK (PDB ID: 4FL2), (D) isoferulic acid with the P2RY12 receptor (PDB ID: 4NTJ), and (E) kaempferol-3-glucuronide with PLCγ2 (PDB ID: 8T7C). Hydrogen bonds, hydrophobic interactions, π-interactions, and other non-covalent contacts stabilizing the ligand–protein complexes are depicted, with interacting amino acid residues identified by their residue names and sequence numbers with the default setting; Table S1, Compounds identified in the ethanolic extract of *Ocimum sanctum* leaves (EEOSL) with GC-MS analysis. (RT) retention time; (MF) molecular formula; Table S2, (a). Compounds identified in the ethanolic extract of *Ocimum sanctum* leaves (EEOSL) with HR LC-MS analysis (positive ESI). (RT) retention time; (MF) molecular formula; (b) Compounds identified in the ethanolic extract of *Ocimum sanctum* leaves (EEOSL) with HR LC-MS analysis (negative ESI). (RT) retention time; (MF) molecular formula; Table S3, (Molecular docking binding affinities (binding energy, kcal/mol) of major phytochemicals identified in the ethanolic extract of *Ocimum sanctum* (EEOSL) against platelet activation-associated target proteins including glycoprotein VI (GPVI; PDB ID: 2GI7), spleen tyrosine kinase (SYK; PDB ID: 4FL2), phospholipase Cγ2 (PLCγ2; PDB ID: 8T7C), and the purinergic receptor (P2RY12; PDB ID: 4NTJ), and phosphoinositide 3-kinase β (PI3Kβ; UniProt ID: P42338). Binding energies are expressed as (kcal/mol), where more negative values indicate stronger predicted affinities. “Did not bind” represents no stable docking pose was obtained under the predefined docking conditions.

## Abbreviations

The following abbreviations are used in this manuscript:

ADP	Adenosine diphosphate
ATP	Adenosine triphosphate
BSA	Bovine serum albumin
CAT	Catalase
CCl_4_	Carbon tetrachloride
EEOSL	Ethanolic extract of *Ocimum sanctum* leaves
FSC	Forward scatter
GPVI	Glycoprotein VI
GPx	Glutathione peroxidase
H	Hour
H & E	Hematoxylin and Eosin
IP3	Inositol 1,4,5-trisphosphate
LDH	Lactate dehydrogenase
MAPK	Mitogen-activated protein kinase
mg/mL	milligram/milliliter
mL/kg	milliliter/kilogram
MD	Molecular dynamics
mM	Millimolar
NBF	Neutral Buffered Formalin
NF-κB	Nuclear factor kappa B
NSAIDs	Non-steroidal anti-inflammatory drugs
PAGE	Polyacrylamide gel electrophoresis
PRP	Platelet-rich plasma
Rg	Radius of gyration
ROS	Reactive oxygen species
Rpm	Revolution per minute
SALP	Serum glutamate alkaline phosphate
SGOT	Serum glutamate oxaloacetate transaminase
SGPT	Serum glutamate pyruvate transaminase
SOD	Superoxide dismutase
SSC	Side scatter
V	Voltage
μg/mL	microgram/milliliter
