# Supplementary material for: Protective Effects of the Ethanolic Leaf Extract of Ocimum sanctum L. on Collagen-Induced Human Platelet Aggregation and CCl4-Induced Hepatotoxicity in Rats: Molecular Docking Analysis of Platelet Activation Signaling Targets
Source: Molecules. 2026 Aug 7;31(16):2749. doi: 10.3390/molecules31162749 (PMC13515578; doi:10.3390/molecules31162749)
Supplement: Supplementary file 1 [file molecules-31-02749-s001.zip › molecules-4416906-supplementary.pdf]

Supplementary materials

Protective Effects of the Ethanolic Leaf Extract of *Ocimum sanctum* L. on Collagen-Induced Human Platelet aggregation and CCl_4-Induced Hepatotoxicity in Rats: Molecular Docking Analysis of Platelet Activation Signaling Targets

Mohammad Maaz[1], Agilan Balupillai[2], Saravanabhavan Periyakali[3], Jasmin Padhan[4], Kumar Kalavathy Murugan [4], Joen-Rong Sheu[5*], Jayakumar Thanasekaran[1*]

[1]Environmental Toxicology and Molecular Phytotherapy Lab, Department of Ecology and Environmental Sciences, School of Life Sciences, Pondicherry University, Puducherry 605014, India

[2]Department of Biotechnology, School of Life Sciences, Pondicherry University, Puducherry 605014, India

[3]Department of Zoology, Bharathiar University, Coimbatore 641046, Tamil Nadu, India

[4]Department of Bioinformatics, School of Life Sciences, Pondicherry University, Puducherry 605014, India

[5]Graduate Institute of Medical Sciences, College of Medicine, Taipei Medical University, Taipei 110, Taiwan

*Correspondence: tjayakumar@pondiuni.ac.in (T.J); Tel.: +91-413-2655325 (ext.325); sheujr@tmu.edu.tw (J.-R.S.); Tel.: +886-2-27361661 (ext. 3199); Fax: +886-2-27390450

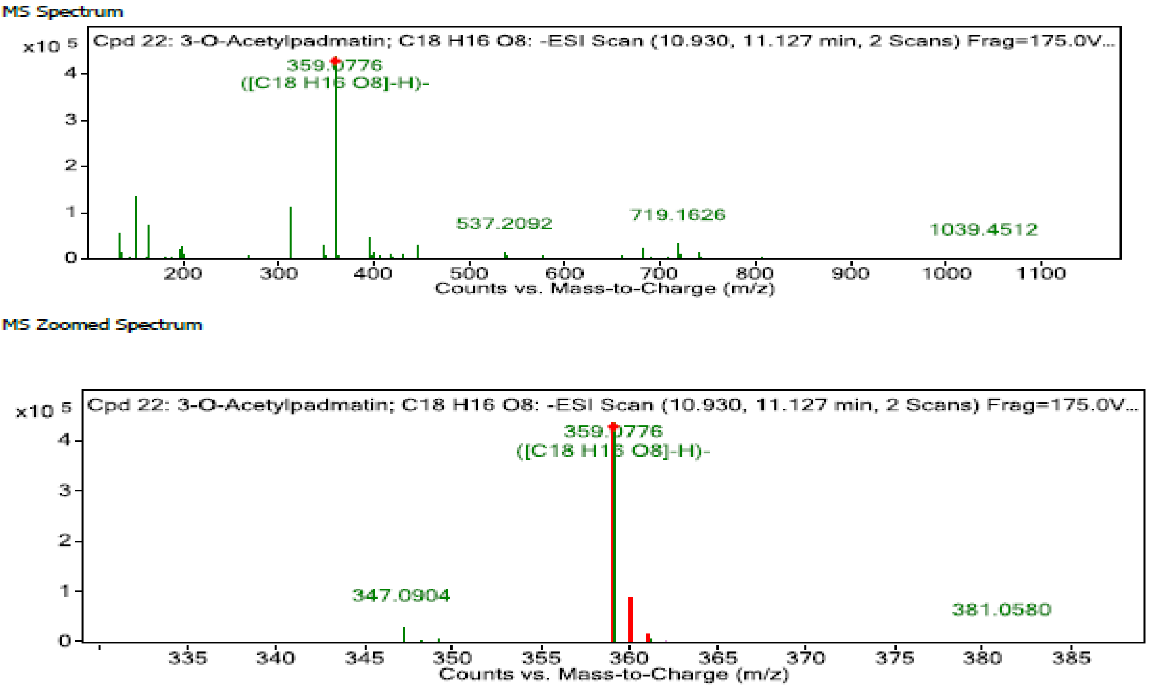

Supplementary Figure S1. Regular and expanded mass spectrum of 3-O-acetylpadmatin identified by HR LC-MS analysis of ethanolic extract of *Ocimum sanctum* leaves (EEOSL).

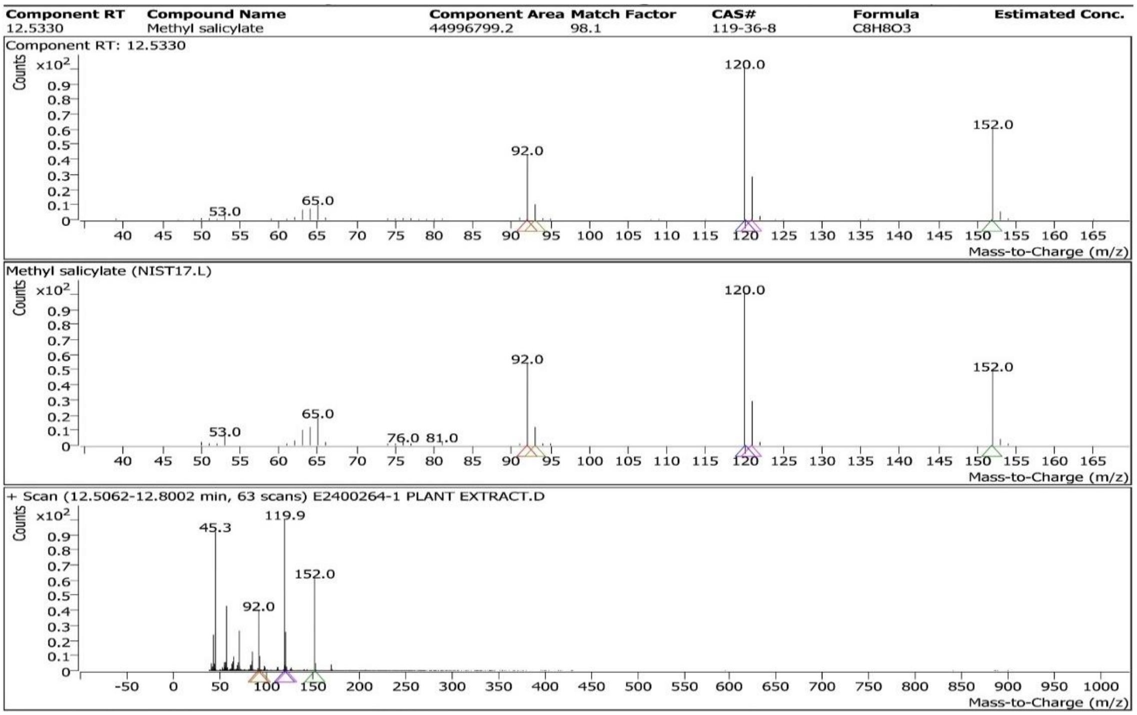

Supplementary Figure S2. Regular mass spectrum of methyl salicylate identified from GC- MS/MS analysis of ethanolic extract of *Ocimum sanctum* leaves (EEOSL).

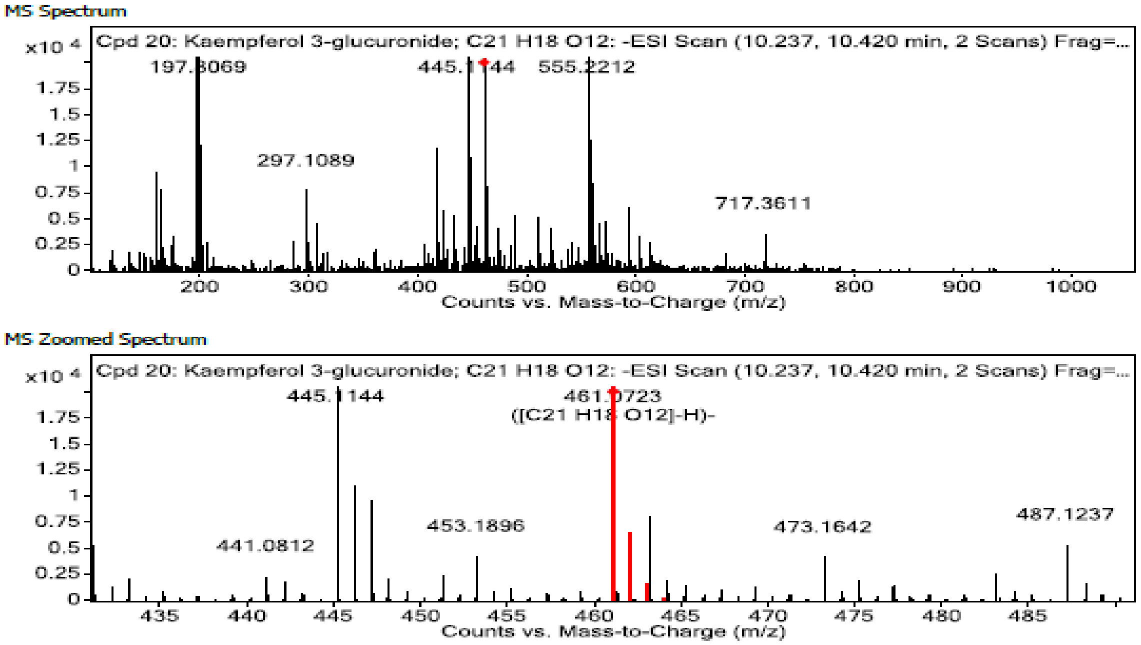

Supplementary Figure S3. Regular and expanded mass spectrum of kaempferol 3-glucuronide identified by HR LC-MS analysis of ethanolic extract of *Ocimum sanctum* leaves (EEOSL).

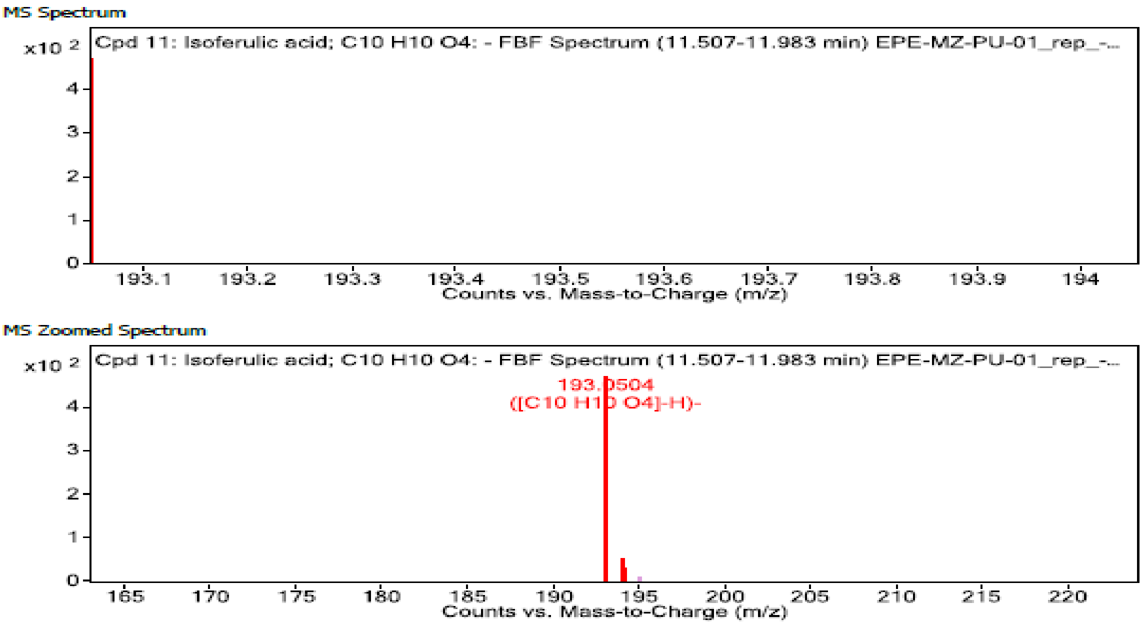

Supplementary Figure S4. Regular and expanded spectrum of isoferulic acid identified by HRLC-MS analysis of ethanolic extract of *Ocimum sanctum* leaves (EEOSL).

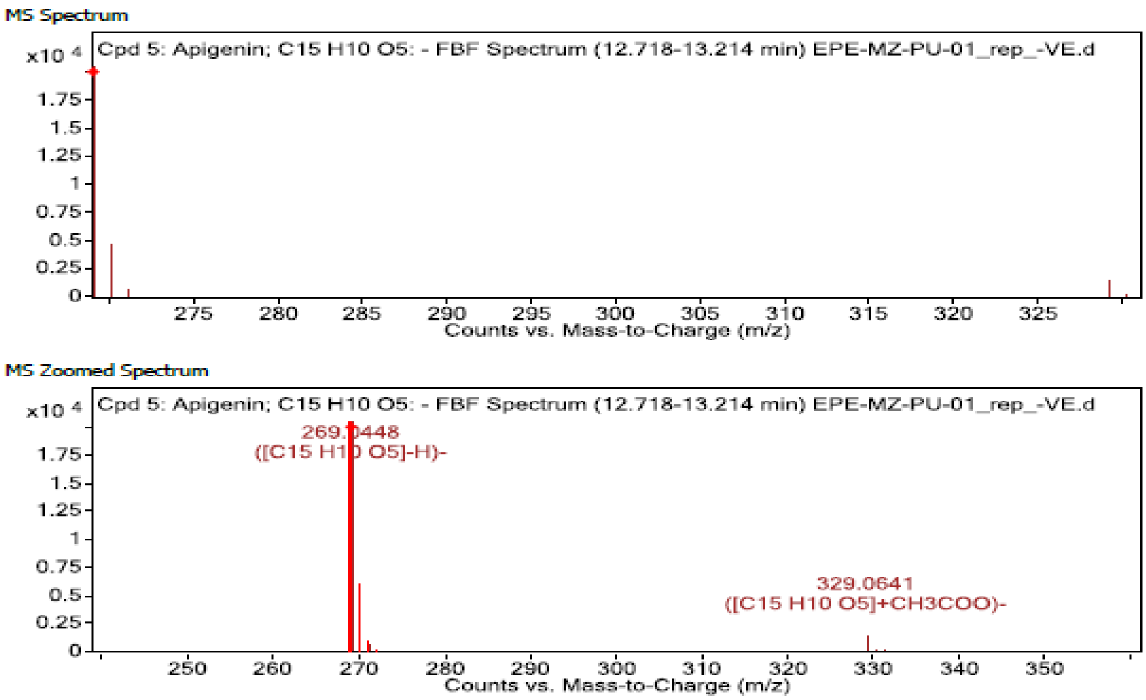

Supplementary Figure S5. Regular and expanded spectrum of apigenin identified by HRLC-MS analysis of ethanolic extract of *Ocimum sanctum* leaves (EEOSL).

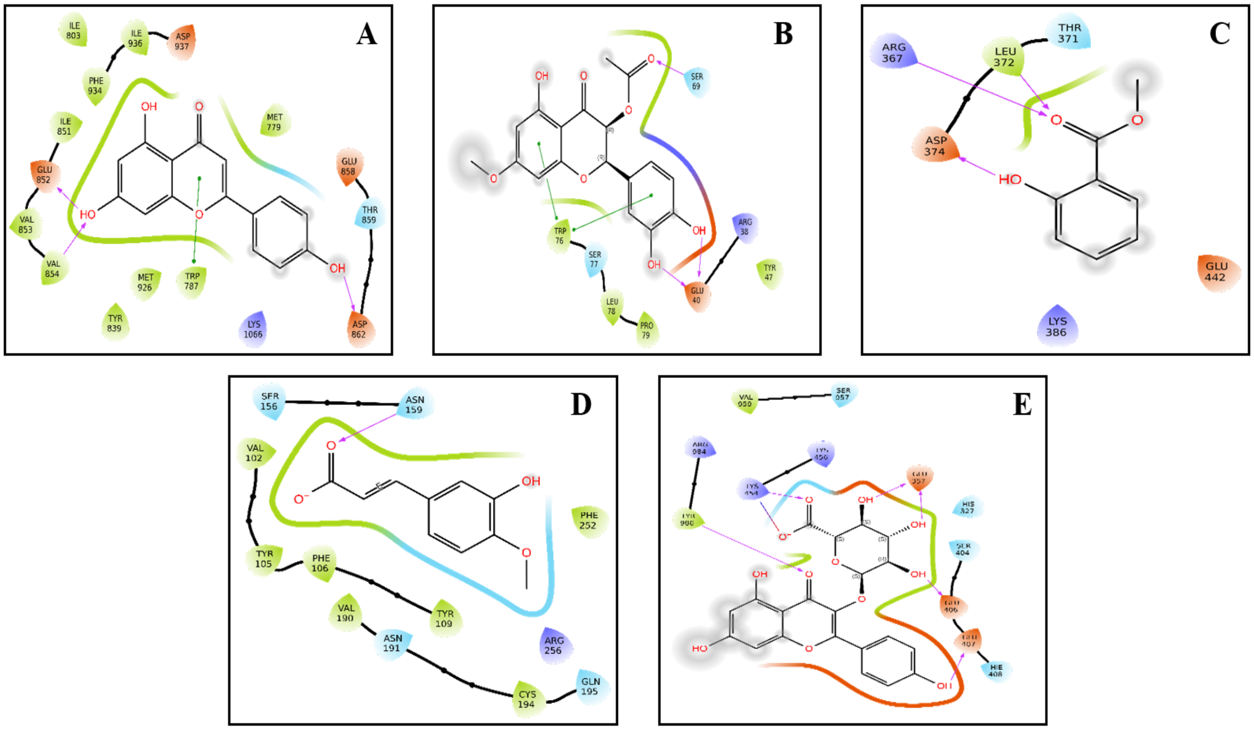

Supplementary Figure S6. Two-dimensional (2D) protein–ligand interaction diagrams generated using the "Ligand Preparation" option in Maestro, illustrating the binding interactions of (A) apigenin with PI3Kβ (UniProt: P42338), (B) 3-O-acetylpadmatin with GPVI (PDB ID: 2GI7), (C) methyl salicylate with SYK (PDB ID: 4FL2), (D) isoferulic acid with the P2RY12 receptor (PDB ID: 4NTJ), and (E) kaempferol-3-glucuronide with PLCγ2 (PDB ID: 8T7C). Hydrogen bonds, hydrophobic interactions, π-interactions, and other non-covalent contacts stabilizing the ligand–protein complexes are depicted, with interacting amino acid residues identified by their residue names and sequence numbers using the default settings.

S. No.	RT	Compound Name	MF
1.	3.1290	3-Pyrrolidinecarboxylic acid, 5-oxo-1-(2-pyridinylmethyl)-	$C_{11}H_{12}N_2O_3$
2.	3.1692	2-Propanol, 1-methoxy-	$C_4H_{10}O_2$
3.	3.2718	2-Propanol, 1-methoxy-	$C_4H_{10}O_2$
4.	3.5057	2-Propanol, 1-methoxy-	$C_4H_{10}O_2$
5.	3.8418	Acetic acid, methoxy-	$C_3H_6O_3$
6.	3.8950	Isobutane	C_4H_{10}
7.	3.9465	Silanol, ethyldimethyl-	$C_4H_{12}OSi$
8.	3.9479	Silanol, dimethyl-	C_2H_{8Osi}
9.	4.1467	Tetraethyl ammonium fluoride	$C_8H_{20}FN$
10.	4.2849	Phenacylidene diacetate	$C_{12}H_{12}O_5$
11.	4.3199	Oxetane, 3-(1-methylethyl)-	$C_6H_{12}O$
12.	4.3414	Sulphuric acid dibutyl ester	$C_8H_{18}O_4S$
13.	4.8549	Silanol, trimethyl-	C_3H_0OSi
14.	4.8553	Ethanol, 2-(trimethylsilyl)-	$C_5H_{14}Osi$
15.	7.0817	2-Hexyl-2-[5-(thiophen-2-yl)thiophen-2-yl]-1,3-dioxolane	$C_{17}H_{22}O_2S_2$
16.	7.7450	Decane	$C_{10}H_{22}$
17.	7.8039	Hexanoic acid, ethyl ester	$C_8H_{16}O_2$
18.	8.1603	Benzyl chloride	C_7H_7Cl
19.	8.4431	4-Decene, 2,2-dimethyl-, (Z)-	$C_{12}H_{24}$
20.	10.2513	Propane, 1,1,3,3-tetraethoxy-	$C_{11}H_{24}O_4$
21.	11.3455	l-Norvaline, N-ethoxycarbonyl-, octyl ester	$C_{16}H_{31}NO_4$
22.	11.3459	4H-Pyran-4-one, 2,3-dihydro-3,5-dihydroxy-6-methyl-	$C_6H_8O_4$
23.	11.3603	(2-Aziridinylethyl)amine	$C_4H_{10}N_2$
24.	11.4916	Cyclopentasiloxane, decamethyl-	$C_{10}H_{30}O_5Si_5$
25.	11.7681	Bicyclo[2.2.1]heptan-2-ol, 1,7,7-trimethyl-, (1Sendo)-	$C_{10}H_{18}O$
26.	12.0780	Valeric acid, .alpha.-methylbenzyl ester	$C_{13}H_{18}O_2$
27.	12.0812	Benzoic acid 2-bromoethyl ester	$C_9H_9BrO_2$
28.	12.1033	Benzoic acid 2-bromoethyl ester	$C_9H_9BrO_2$
29.	12.5330	Methyl salicylate	$C_8H_8O_3$
30.	12.6053	Methylamine, N-(1-ethylhexylidene)-	C_9H_{19N}
31.	14.0307	Naphthalene, 1,2,3,4-tetrahydro-1,1,6-trimethyl-	$C_{13}H_{18}$
32.	14.3606	1,2-Propanediol, 3-(1-pyrrolidinyl)-	$C_7H_{15}NO_2$
33.	14.5212	3,4-Dihydroisoquinolin-7-ol, 1-[4-hydroxybenzyl]-6-methoxy-	$C_{17}H_{17}NO_3$
34.	14.9859	L-Proline, 1-acetyl-	$C_7H_{11}NO3$
35.	15.4964	Naphthalene, 1,2,3,4-tetrahydro-1,6-dimethyl-4-(1-methylethyl)-, (1S-cis)-	$C_{15}H_{22}$
36.	15.9996	Naphthalene, 1,2,3,4-tetrahydro-1,6,8-trimethyl-	$C_{13}H_{18}$
37.	16.7409	(1S,4S,4aS)-1-Isopropyl-4,7-dimethyl-1,2,3,4,4a,5-hexahydronaphthalene	$C_{15}H_{24}$
38.	16.7916	Naphthalene, 1,2,3,4-tetrahydro-1,6,8-trimethyl-	$C_{13}H_{18}$
39.	16.8361	Naphthalene, 1,2-dihydro-1,1,6-trimethyl-	$C_{13}H_{16}$
40.	17.0443	Eugenol	$C_{10}H_{12}O_2$
41.	17.4007	Cubenene	$C_{15}H_{24}$

42.	17.4039	(1S,4S,4aS)-1-Isopropyl-4,7-dimethyl-1,2,3,4,4a,5-hexahydronaphthalene	$C_{15}H_{24}$
43.	17.5620	1-Octylsilatrane	$C_{14}H_{29}NO_3Si$
44.	17.6186	(-)-.beta.-Bourbonene	$C_{15}H_{24}$
45.	17.6622	Nonane, 1,1-diethoxy-	$C_{13}H_{28}O_2$
46.	17.7962	Cyclohexane, 1-ethenyl-1-methyl-2,4-bis(1-methylethenyl)-, [1S-(1.alpha.,2.beta.,4.beta.)]-	$C_{15}H_{24}$
47.	17.8274	1, 1, 5-Trimethyl-1, 2-dihydronaphthalene	$C_{13}H_{16}$
48.	17.9566	Tetradecane	$C_{14}H_{30}$
49.	18.0767	Sulfoxide, methyl phenethyl	$C_9H_{12}OS$
50.	18.0799	Methyleugenol	$C_{11}H_{14}O_2$
51.	18.2239	p-Anisic acid, 4-nitrophenyl ester	$C_{11}H_{14}NO_5$
52.	18.2267	Methyleugenol	$C_{14}H_{11}NO5$
53.	18.2269	Benzene, 1,2-dimethoxy-4-(1-propenyl)-	$C_{11}H_{14}O_2$
54.	18.2751	Methyleugenol	$C_{11}H_{14}O_2$
55.	18.3240	p-Cyanophenyl p-(3-butenyloxy)benzoate	$C_{18}H_{15}NO_3$
56.	18.4145	Bicyclo[5.2.0]nonane, 2-methylene-4,8,8-trimethyl-4-vinyl-	$C_{15}H_{24}$
57.	18.5978	Benzene, 1,4-bis(1-formylethyl)-	$C_{12}H_{14}O_2$
58.	18.7089	2-Phenylacetamide, N-(1-phenyl-2-propyl)-	$C_{17}H_{19}NO$
59.	18.7100	cis-.alpha.-Bergamotene	$C_{15}H_{24}$
60.	18.8537	(3aR,4R,8R,8aS)-3a,4,8a-Trimethyl-7-methylenedecahydro-4,8-methanoazulene-rel-	$C_{15}H_{24}$
61.	19.0593	1,4,7,-Cycloundecatriene, 1,5,9,9-tetramethyl-,Z,Z,Z-	$C_{15}H_{24}$
62.	19.2287	Benzenamine, 2,5-dichloro-	
63.	19.4524	3a,7-Methano-3aH-cyclopentacyclooctene,1,4,5,6,7,8,9,9a-octahydro-1,1,7-trimethyl-, [3aR-(3a.alpha.,7.alpha.,9a.beta.)]-	$C_{15}H_{24}$
64.	19.4531	Naphthalene, decahydro-4a-methyl-1-methylene-7-(1-methylethylidene)-, (4aR-trans)-	$C_{15}H_{24}$
65.	19.4800	1,2-Benzenediol, O-(4-butylbenzoyl)-O'-(isobutoxycarbonyl)-	$C_{22}H_{26}O_5$
66.	19.5620	Germacrene D	$C_{15}H_{24}$
67.	19.6530	1,5-Cyclodecadiene, 1,5-dimethyl-8-(1-methylethenyl)-, [S-(Z,E)]-	$C_{15}H_{24}$
68.	19.6536	Naphthalene, decahydro-4a-methyl-1-methylene-7-(1-methylethenyl)-, [4aR-(4a.alpha.,7.alpha.,8a.beta.)]-	$C_{15}H_{24}$
69.	19.8037	Naphthalene, decahydro-4a-methyl-1-methylene-7-(1-methylethylidene)-, (4aR-trans)-	$C_{15}H_{24}$
70.	19.9195	7-Isoquinolinol, 1,2,3,4-tetrahydro-6-methoxy-1-salicyl-	$C_{17}H_{19}NO_3$
71.	19.9437	Spiro[5.5]undeca-1,8-diene, 1,5,5,9-tetramethyl-,(R)-	$C_{15}H_{24}$
72.	19.9921	Benzene, 1-methyl-4-(1,2,2-trimethylcyclopentyl)-,(R)-	$C_{15}H_{22}$
73.	20.0967	2,4-Di-tert-butylphenol	$C_{14}H_{22}O$
74.	20.1060	Benzene, 1-(1,1-dimethylethyl)-3-ethyl-5-methyl-	$C_{13}H_{20}$
75.	20.2611	Naphthalene, 1,2,3,5,6,8a-hexahydro-4,7-dimethyl-1-(1-methylethyl)-, (1S-cis)-	$C_{15}H_{24}$

76.	20.4031	1H-Benzocycloheptene, 2,4a,5,6,7,8-hexahydro-3,5,5,9-tetramethyl-, (R)-	$C_{15}H_{24}$
77.	20.4037	(Z)-1-Methyl-4-(6-methylhept-5-en-2-ylidene)cyclohex-1-ene	$C_{15}H_{24}$
78.	20.5804	4-Isopropyl-6-methyl-1-methylene-1,2,3,4-tetrahydronaphthalene	$C_{15}H_{20}$
79.	20.6154	1,2-Benzenediol, O-(5-chlorovaleryl)-O'-(1-naphthoyl)-	$C_{22}H_{19}ClO_4$
80.	20.6561	1,2-Cyclohexanedicarboxylic acid, difurfuryl ester	$C_{18}H_{28}O_6$
81.	20.6837	Cyclohexanemethanol, 4-ethenyl-.alpha.,.alpha.,4-trimethyl-3-(1-methylethenyl)-, [1R-(1.alpha.,3.alpha.,4.beta.)]-	$C_{15}H_{26}O$
82.	21.1515	2H-1-benzopyran-2-one, 3,4-dihydro-4,4,6-trimethyl-	$C_{12}H_{14}O_2$
83.	21.2099	Caryophyllene oxide	$C_{15}H_{24}O$
84.	21.3653	Diethyl Phthalate	$C_{12}H_{14}O_4$
85.	21.6480	Cycloocta-1,3,6-triene, 2,3,5,5,8,8-hexamethyl-	$C_{14}H_{22}$
86.	21.6822	l-Alanine, N-(2,3,4-trifluorobenzoyl)-, methyl ester	$C_{11}H_{10}F_3NO_3$
87.	21.8097	L-Alanine, 2-methyl-N-(n-propyl)-N-(4-methylphenyl)-, n-propyl ester	$C_{17}H_{27}NO_2$
88.	21.8097	Megastigmatrienone	$C_{13}H_{18}O$
89.	21.8850	Ethanone, 1,1',1''-(1,3,5-benzenetriyl)tris-	$C_{12}H_{12}O_3$
90.	21.9558	Tetracyclo[6.3.2.0(2,5).0(1,8)]tridecan-9-ol, 4,4-dimethyl-	$C_{15}H_{24}O$
91.	21.9953	D-Alanine, N-(4-butylbenzoyl)-, heptyl ester	$C_{21}H_{33}NO_3$
92.	22.1433	Phenol, 4-(1,1-dimethylethyl)-2-methyl-	$C_{11}H_{16}O$
93.	22.2009	Selin-6-en-4.alpha.-ol	$C_{15}H_{26}O$
94.	22.4506	1H-3a,7-Methanoazulen-6-ol, octahydro-3,6,8,8-tetramethyl-, acetate, [3R-(3.alpha.,3a.beta.,6.alpha.,7.beta.,8a.alpha.)]-	$C_{17}H_{28}O_2$
95.	22.6166	3-(p-Fluorobenzoyl)-propionic acid	$C_{10}H_9FO_3$
96.	22.6682	2'-Hydroxyacetophenone, TMS derivative	$C_{11}H_{16}O_2Si$
97.	23.5807	Benzyl Benzoate	$C_{14}H_{12}O_2$
98.	23.5874	Ethanone, 2-chloro-1,2-diphenyl-	$C_{14}H_{11}ClO$
99.	23.7535	1-Dodecanol, 2-hexyl-	$C_{18}H_{38}O$
100.	23.9321	Cyclohexane, decyl-	$C_{16}H_{32}$
101.	24.2737	2-Hexadecen-1-ol, 3,7,11,15-tetramethyl-, acetate,[R-[R*,R*-(E)]]-	$C_{22}H_{42}O_2$
102.	24.3543	3,7,11,15-Tetramethylhexadec-2-ene	$C_{20}H_{40}$
103.	24.5417	Neophytadiene	$C_{20}H_{38}$
104.	24.5461	2-Fluorobenzylamine, N,N-didecyl	$C_{27}H_{48}FN$
105.	54.6521	Dibutyl phthalate	$C_{16}H_{22}O_4$
106.	24.7361	2-Hexadecen-1-ol, 3,7,11,15-tetramethyl-, acetate,[R-[R*,R*-(E)]]-	$C_{22}H_{42}O_2$
107.	25.0541	Lidocaine	$C_{14}H_{22}N_2O$
108.	25.6539	n-Hexadecanoic acid	$C_{16}H_{32}O_2$
109.	25.6719	5-(4-Hexyloxybenzoyloxy)-2-(4-nitrophenyl)pyrimidine	$C_{23}H_{23}N_3O_5$
110.	25.6754	Dibutyl phthalate	$C_{16}H_{22}O_4$
111.	25.9837	Hexadecanoic acid, ethyl ester	$C_{18}H_{36}O_2$

112.	26.1872	Benzenepropanoic acid, 3,5-bis(1,1-dimethylethyl)-4-hydroxy-, ethyl ester	$C_{19}H_{30}O_3$
113.	27.3909	Phytol	$C_{20}H_{40}O$
114.	27.7345	9,12,15-Octadecatrienoic acid, (Z,Z,Z)-	$C_{18}H_{30}O_2$
115.	27.9233	Linoleic acid ethyl ester	$C_{20}H_{36}O_2$
116.	27.9354	Octadecanoic acid	$C_{18}H_{36}O_2$
117.	28.0009	9,12,15-Octadecatrienoic acid, ethyl ester, (Z,Z,Z)-	$C_{20}H_{34}O_2$
118.	28.2450	Octadecanoic acid, ethyl ester	$C_{20}H_{40}O_2$
119.	28.2810	Docosane	$C_{22}H_{46}$
120.	28.9573	Benzoic acid, 2-methylpropyl ester	$C_{11}H_{14}O_2$
121.	28.5155	3,7,11,15-Tetramethylhexadec-2-en-1-yl acetate	$C_{22}H_{42}O_2$
122.	29.1637	Acetic acid, (dodecahydro-7-hydroxy-1,4b,8,8-tetramethyl-10-oxo-2(1H)-phenanthrenylidene)-,2-(dimethylamino)ethyl ester	$C_{24}H_{39}NO_4$
123.	29.3981	Benzoic acid, 2-methylpropyl ester	$C_{11}H_{14}O_2$
124.	30.2417	Hexanedioic acid, bis(2-ethylhexyl) ester	$C_{22}H_{42}O_4$
125.	30.7760	Benzphetamine	$C_{17}H_{21}N$
126.	30.7783	N-Ethyl-N-benzylglycine ethyl ester	$C_{13}H_{19}NO_2$
127.	31.2362	Hexadecanoic acid, 2-hydroxy-1-(hydroxymethyl)ethyl ester	$C_{19}H_{38}O_4$
128.	31.2793	Bis(pentamethylcyclopentadienyl)iron	$C_{20}H_{30}Fe$
129.	31.4472	Benzyldiethyl-(2,6-xylylcarbamoylmethyl)-ammonium benzoate	$C_{28}H_{34}N_2O_3$
130.	31.5537	Phthalic acid, di(2-propylpentyl) ester	$C_{24}H_{38}O_4$
131.	32.5960	Octocrylene	$C_{24}H_{27}NO_2$
132.	32.6857	Heptacosane	$C_{27}H_{56}$
133.	32.7499	Silane, diethyl(cis-4-methylcyclohexyloxy)undecyloxy-	$C_{22}H_{46}O_2Si$
134.	32.8847	Octadecanoic acid, 2,3-dihydroxypropyl ester	$C_{21}H_{42}O_4$
135.	33.0918	Isophthalic acid, di(4-octyl) ester	$C_{24}H_{38}O_4$
136.	33.7549	Supraene	$C_{30}H_{50}$
137.	34.0220	Silane, diethylhexadecyloxy(2-phenylethoxy)-	$C_{28}H_{52}O_2Si$
138.	34.0222	Silane, diethylpentadecyloxy(3-phenylpropoxy)-	$C_{28}H_{52}O_2Si$
139.	34.2138	Silane, diethylpentadecyloxy(3-phenylpropoxy)-	$C_{28}H_{52}O_2Si$
140.	34.2196	.alpha.-Tocospiro A	$C_{29}H_{50}O_4$
141.	34.3094	Nonacosane	$C_{29}H_{60}$
142.	34.7334	1,6,10,14,18,22-Tetracosahexaen-3-ol,2,6,10,15,19,23-hexamethyl-, (all-E)-	$C_{30}H_{50}O$
143.	35.3096	Hexane, 3-ethyl-3-methyl-	C_9H_{20}
144.	35.3125	Triacontane	$C_{30}H_{62}$
145.	35.6071	1,6,10,14,18,22-Tetracosahexaen-3-ol,2,6,10,15,19,23-hexamethyl-, (all-E)-	$C_{30}H_{50}O$
146.	35.6151	2-Methyl-3-(3-methyl-but-2-enyl)-2-(4-methyl-pent-3-enyl)-oxetane	$C_{15}H_{26}O$
147.	36.0711	.beta.-Tocopherol	$C_{28}H_{48}O_2$
148.	36.0723	.gamma.-Tocopherol	$C_{28}H_{48}O_2$
149.	36.5124	Tetratriacontane	$C_{34}H_{70}$

150.	36.9814	Cholest-5-en-3-ol, (3.alpha.)-	$C_{27}H_{46}O$
151.	37.1487	dl-.alpha.-Tocopherol	$C_{29}H_{50}O_2$
152.	37.1572	1,4-Bis-(4-fluorophenylethynyl)-2,3,5,6-tetrafluorobenzene	$C_{22}H_8F_6$
153.	37.5691	Hentriacontane, 3-methyl-	$C_{32}H_{66}$
154.	37.6198	2,6-Bis(3,4-methylenedioxyphenyl)-3,7-dioxabicyclo(3.3.0)octane	$C_{20}H_{18}O_6$
155.	37.9468	Thiophen-2-methylamine, N-(2-fluorophenyl)-	$C_{11}H_{10}FNS$

Supplementary Table S1. Compounds identified in the ethanolic extract of *Ocimum sanctum* leaves (EEOSL) by GC-MS analysis. (RT) retention time; (MF) molecular formula.

S. No.	RT	Compound Name	MF	Mass
1.	1.316	Proline betaine	$C_7H_{14}NO_2$	144.1015
2.	3.142	Caproylcholine	$C_{11}H_{24}NO_2$	202.1791
3.	9.29	Valyl-Tyrosine	$C_{14}H_{20}N_2O_4$	280.1418
4.	11.322	Triamterene	$C_{12}H_{11}N_7$	253.1076
5.	12.074	b-D-Glucopyranosiduronic acid, 3-(6-hydroxy-2-naphthalenyl)-1-methylpropyl	$C_{20}H_{24}O_8$	392.1469
6.	12.303	alpha-Obscurine	$C_{17}H_{26}N_2O$	274.2038
7.	12.388	His-His-OH	$C_{18}H_{18}N_6O_6$	414.1275
8.	12.626	2alpha,7beta,15beta,18- tetraacetoxy-cholest-5-en-3alpha-ol	$C_{35}H_{54}O_9$	618.3706
9.	12.86	Benztropine	$C_{21}H_{25}NO$	307.1917
10.	12.862	Nigrifactin	$C_{12}H_{17}N$	175.1346
11.	13.006	Albine	$C_{14}H_{20}N_2O$	232.1552
12.	13.166	M 344	$C_{16}H_{25}N_3O_3$	307.1912
13.	13.256	C16 Sphingosine-1-phosphate	$C_{16}H_{34}NO_5P$	351.2159
14.	13.514	Ozagrel	$C_{13}H_{12}N_2O_2$	228.0894
15.	14.342	(+)-6-Methyl caprylic acid	$C_9H_{18}O_2$	158.1317
16.	14.433	Hirsutidin	$C_{18}H_{17}O_7$	345.0939
17.	14.697	(1R,2R)-3-oxo-2-pentyl- cyclopentanehexanoic acid	$C_{16}H_{28}O_3$	268.2034
18.	14.845	D-Linalool 3-(6''- malonylglucoside)	$C_{19}H_{30}O_9$	402.1892
19.	14.995	(S)-2,3-Dihydro-3,5-dihydroxy- 2-oxo-3-indoleacetic acid 5- glucoside	$C_{16}H_{19}NO_{10}$	385.1003
20.	15.83	Fluprostenol Lactone Diol	$C_{18}H_{19}F_3O_5$	372.1165
21.	16.218	Mitoxantrone	$C_{22}H_{28}N_4O_6$	444.2008
22.	16.503	Diethylpropion(metabolite V- glucuronide)	$C_{19}H_{27}NO_8$	397.1736
23.	16.517	Arg Trp Ala	$C_{20}H_{29}N_7O_4$	431.2266
24.	17.133	3-Hydroxymugineic acid	$C_{12}H_{20}N_2O_9$	336.1192
25.	17.19	Racemosin	$C_{16}H_{16}O_5$	288.0988
26.	17.842	Limonoate	$C_{26}H_{34}O_{10}$	506.2152
27.	18.183	N-(3S-hydroxydecanoyl)-L- serine	$C_{13}H_{25}NO_5$	275.1741
28.	18.587	Terminaline	$C_{23}H_{41}NO_2$	363.3102
29.	18.594	Tranylcypromine glucuronide	$C_{15}H_{19}NO_6$	309.1211
30.	19.744	Cetrimonium	$C_{19}H_{42}N$	284.3287
31.	20.078	N,N,N-trimethyl-sphingosine	$C_{21}H_{44}NO_2$	342.3328
32.	21.381	(-)-Batrachotoxinin A	$C_{24}H_{35}NO_5$	417.2503
33.	21.593	6β-Hydroxyestradiol-17β	$C_{18}H_{24}O_3$	288.1711
34.	21.625	22α-Hydroxy-5α-campestan-3- one	$C_{28}H_{48}O_2$	416.3652
35.	21.887	PtdIns-(1,2-dioctanoyl)	$C_{25}H_{47}O_{13}P$	586.2754
36.	22.423	Khivorin	$C_{32}H_{42}O_{10}$	586.2764
37.	22.508	Swietenine	$C_{32}H_{40}O_9$	568.2655
38.	22.77	Ramipril glucuronide	$C_{29}H_{40}N_2O_{11}$	592.2632
39.	22.991	10Z-Pentacosene	$C_{25}H_{50}$	350.3874
40.	23.215	Trandolapril glucuronide	$C_{30}H_{42}N_2O_{11}$	606.2778

Supplementary Table S2. (a) Compounds identified in the ethanolic extract of *Ocimum sanctum* leaves (EEOSL) by HR LC-MS analysis (positive ESI). (RT) retention time; (MF) molecular formula.

S. No.	RT	Compound Name	MF	Mass
1.	3.053	Ascorbic Acid	$C_6H_8O_6$	176.0309
2.	8.732	Eudesmic acid	$C_{10}H_{12}O_5$	212.0678
3.	9.289	Caffeic acid	$C_9H_8O_4$	180.0415
4.	10.06	Resokaempferol 3-glucoside	$C_{21}H_{20}O_{10}$	432.1059
5.	10.152	Biochanin A 7-O-rutinoside	$C_{28}H_{32}O_{14}$	592.1787
6.	10.294	Quercitrin	$C_{21}H_{20}O_{11}$	448.1015
7.	10.304	Isoprunetin 7-O-β-D- glucopyranoside	$C_{22}H_{22}O_{10}$	446.1207
8.	10.351	Kaempferol 3-glucuronide	$C_{21}H_{18}O_{12}$	462.0829
9.	10.42	Chlorogenic acid	$C_{16}H_{18}O_9$	354.094
10.	10.938	Pyrazosulfuron	$C_{12}H_{14}N_6O_7S$	386.0651
11.	10.989	Cirsimaritin	$C_{17}H_{14}O_6$	314.0793
12.	11.041	3-O-Acetylpadmatin	$C_{18}H_{16}O_8$	360.0848
13.	11.048	Dehydrogriseofulvin	$C_{17}H_{15}ClO_6$	350.0552
14.	11.094	Xanthochymuside	$C_{36}H_{32}O_{16}$	720.1707
15.	11.1	S-(Hydroxymethyl)mycothiol	$C_{18}H_{32}N_2O_{13}S$	516.1628
16.	11.105	Kaempferol 3-(6''- malonylglucoside)-7-glucoside	$C_{30}H_{32}O_{19}$	696.1466
17.	11.233	Azelaic acid	$C_9H_{16}O_4$	188.104
18.	11.308	Dehydrogriseofulvin	$C_{17}H_{15}ClO_6$	350.0542
19.	11.383	Quercetagetin 7,3',4'- trimethyl ether	$C_{18}H_{16}O_8$	360.0843
20.	11.524	Magnesium protoporphyrin monomethyl ester	$C_{35}H_{34}MgN_4O_4$	598.2433
21.	11.706	S-Formylmycothiol	$C_{18}H_{30}N_2O_{13}S$	514.148
22.	11.764	Fusarenone X	$C_{17}H_{22}O_8$	354.1315
23.	11.858	Isoferulic acid	$C_{10}H_{10}O_4$	194.0577
24.	11.875	Quercetin 3,3'-dimethyl ether 4'-(2-methylbutyrate)	$C_{22}H_{22}O_8$	414.1307
25.	11.964	Cyrtopterin	$C_{23}H_{26}O_{10}$	462.1521
26.	11.966	Medicagenic acid 3-O-beta-D- glucoside	$C_{36}H_{56}O_{11}$	664.3825
27.	11.97	Mikanin 3-galactoside	$C_{24}H_{26}O_{12}$	506.1423
28.	11.983	2'-Hydroxygenistein	$C_{15}H_{10}O_6$	286.0471
29.	12.228	Edulone A	$C_{22}H2_2O_8$	414.1322
30.	12.387	Lys-Gln-Ala-Gly-Asp-Val	$C_{25}H_{44}N_8O_{10}$	616.3154
31.	12.396	Glycosyl-4,4'- diaponeurosporenoate	$C_{36}H_{50}O_7$	594.3525
32.	12.6	Fusarenone X	$C_{17}H_{22}O_8$	354.1309
33.	12.61	Formononetin 7-O-(6''- acetylglcoside)	$C_{24}H_{24}O_{10}$	472.1373
34.	12.792	Apigenin	$C_{15}H_{10}O_5$	270.052
35.	13.06	Dehydrogriseofulvin	$C_{17}H_{15}ClO_6$	350.0531
36.	13.09	Prosapogenin	$C_{36}H_{54}O_{11}$	662.3686
37.	13.196	Quercetin 3-galactoside-7- glucoside	$C_{27}H_{30}O_{17}$	626.1426
38.	13.42	BL V	$C_{22}H_{16}O_9$	424.0788
39.	13.608	Gibberellin A86	$C_{19}H_{24}O_8$	380.1469
40.	13.699	5-Hydroxy-6-methoxy-3',4'-methylenedioxyfurano[2'',3'': 7,8]flavanone	$C_{19}H_{14}O_7$	354.0741
41.	13.948	α- Terpinene	$C_{10}H_{16}$	136.124
42.	14.149	Cirsilineol	$C_{18}H_{16}O_7$	344.0893

43.	14.596	12-Deoxyaklanonic acid	$C_{21}H_{18}O_7$	382.1054
44.	15.533	Arjunolic acid	$C_{30}H_{48}O_5$	488.3501
45.	15.684	Gefitinib	$C_{22}H_{24}ClFN_4O_3$	446.1499
46.	15.982	Juvabione	$C_{16}H_{26}O_3$	266.1872
47.	16.67	all-trans-pentaprenyl diphosphate	$C_{25}H_{44}O_7P_2$	518.2546
48.	16.931	Hoslundal	$C_{18}H_{14}O_5$	310.0836
49.	17.197	Deltaline	$C_{27}H_{41}NO_8$	507.2847
50.	17.217	• LysoPE(0:0/20:3(8Z,11Z,14Z))	$C_{25}H_{46}NO_7P$	503.3002
51.	17.44	• 3-[[5-Methyl-2-(1-methylethyl)cyclohexyl]oxy]- 1,2-propanediol	$C_{13}H_{26}O_3$	230.186
52.	17.517	α-9(10)-EpODE	$C_{18}H_{30}O_3$	294.2192
53.	17.784	Colnelenic acid	$C_{18}H_{28}O_3$	292.2033
54.	17.884	dolichyl diphosphate	$C_{25}H_{46}O_7P_2$	520.2703
55.	17.98	methyl 8-[2-(2-formyl-vinyl)- 3-hydroxy-5-oxo-cyclopentyl]- octanoate	$C_{17}H_{26}O_5$	310.1812
56.	18.048	Sebiferenic acid	$C_{30}H_{48}O_4$	472.3553
57.	18.221	PG(18:2(9Z,12Z)/0:0)	$C_{24}H_{45}O_9P$	508.2807
58.	18.23	LysoPE(0:0/20:2(11Z,14Z))	$C_{25}H_{48}NO_7P$	505.3158
59.	18.315	Fexaramine	$C_{32}H_{36}N_2O_3$	496.2728
60.	18.392	Sebiferenic acid	$C_{30}H_{48}O_4$	472.3555
61.	18.654	G-418	$C_{20}H_{40}N_4O_{10}$	496.272
62.	19.084	LysoPE(0:0/18:0)	$C_{23}H_{48}NO_7P$	481.316
63.	19.089	PG(16:0/0:0)[U]	$C_{22}H_{45}O_9P$	484.2803
64.	20.272	1beta,3beta,5alpha,6beta-tetrahydroxyandrostan-17- one	$C_{19}H_{30}O_5$	338.2037
65.	21.139	Oleanolic acid	$C_{30}H_{48}O_3$	456.3613
66.	21.491	TG(17:2(9Z,12Z)/20:5(5Z,8Z, 11Z,14Z,17Z)/22:6(4Z,7Z,10 Z,13Z,16Z,19Z))[iso6]	$C_{62}H_{94}O_6$	934.7063
67.	21.496	• TG(15:0/20:4(5Z,8Z,11Z,14Z) • /22:6(4Z,7Z,10Z,13Z,16Z,19Z))[iso6]	$C_{60}H_{96}O_6$	912.7227
68.	22.226	6,8a-Seco-6,8a-deoxy-5-oxoavermectin "2b" aglycone	$C_{33}H_{48}O_8$	572.3352
69.	22.404	Prostaglandin D1 Alcohol	$C_{20}H_{36}O_4$	340.2604
70.	23.462	20-HEDE	$C_{20}H_{36}O_3$	324.2656
71.	23.974	Inuline	$C_{32}H_{46}N_2O_8$	586.3258
72.	24.007	Arjungenin	$C_{30}H_{48}O_6$	504.346
73.	24.203	Xanthochymol	$C_{38}H_{50}O_6$	602.3615

Supplementary Table S2. (b) Compounds identified in the ethanolic extract of *Ocimum sanctum* leaves (EEOSL) by HR LC-MS analysis (negative ESI). (RT) retention time; (MF) molecular formula.

Sl. No.	Compound name	Compound CID	GPVI (2GI7)	SYK (4FL2)	PLCγ2 (8T7C)	P2RY12 (4NTJ)	PI3Kβ (Uniport: P42338)
1.	Methyleugenol	7127	-3.41	-3.13	-2.7	-6.05	-4.81
2.	Eugenol	3314	-3.31	-3.35	-3.2	-5.84	-6.06
3.	Oleanolic acid	10494	-1.58	Did not bind	-2.79	Did not bind	-3.07
4.	DL-alpha-tocopherol	2116	-2.03	-3.51	-2.51	-5.46	-5.36
5.	Phytol	5280435	0.81	Did not bind	0.12	-1.78	-1.15
6.	Kaempferol-3-glucuronide	22846027	-2.98	Did not bind	-5.84	-5.4	-6.4
7.	Apigenin	5280443	-4.09	Did not bind	-5.15	-5.76	-8.01
8.	2,4-Di-tert-butylphenol	7311	-4.13	Did not bind	-3.71	Did not bind	-4.33
9.	(+)-6-Methyl caprylic acid	10447	-2.13	Did not bind	-1.63	-3.27	-3.71
10.	Formononetin 7-O-(6''-acetylglcoside)	44257222	-1.99	-2.13	-3.55	-5.43	-4.98
11.	Isoferulic acid	736186	-3.6	Did not bind	-3.47	-6.6	-6.77
12.	3-O-Acetylpadmatin	10406203	-4.61	Did not bind	-5.04	-3.48	-6.01
13.	Methyl salicylate	4133	-4.2	-3.53	-3.74	-5.27	-6.36
14.	Caffeic Acid	689043	-3.62	Did not bind	-4.36	Did not bind	-6.27
15.	Caryophyllene oxide	1742210	Did not bind	Did not bind	-3.12	-3.69	-5.94

Supplementary Table S3. Molecular docking binding affinities (binding energy, kcal/mol) of major phytochemicals identified in the ethanolic extract of *Ocimum sanctum* (EEOSL) against platelet activation-associated target proteins, including glycoprotein VI (GPVI; PDB ID: 2GI7), spleen tyrosine kinase (SYK; PDB ID: 4FL2), phospholipase Cγ2 (PLCγ2; PDB ID: 8T7C), the purinergic receptor (P2Y12; PDB ID: 4NTJ), and phosphoinositide 3-kinase β (PI3Kβ; UniProt ID: P42338). Binding energies are expressed in kcal/mol, where more negative values indicate stronger predicted affinities. "Did not bind" represents no stable docking pose was obtained under the predefined docking conditions.
